# Supplementary material for: Cross-species molecular mapping of the photoreceptor sensory cilium and periciliary structures identifies conserved and species-specific architectural features
Source: J Cell Sci. 2026 Oct 22;139(21):jcs265080. doi: 10.1242/jcs.265080 (PMC13723404; doi:10.1242/jcs.265080)
Supplement: Supplementary file 1 [file joces-139-265080-s1.pdf]

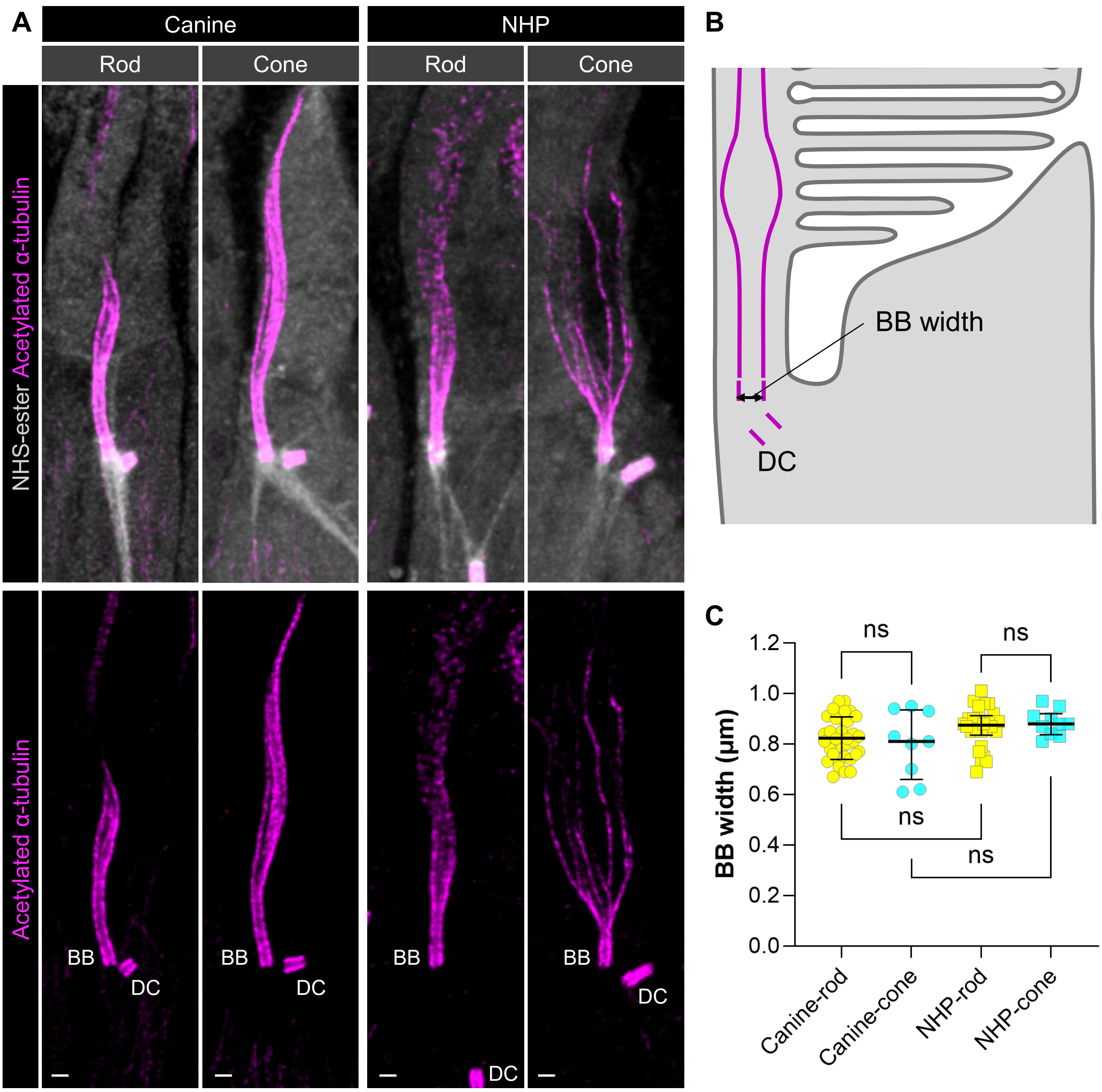

Fig. S1. Comparison of basal body width for determination of the expansion factor.

(A) Representative confocal images of photoreceptor sensory cilium (PSC) architecture in rod and cone photoreceptors from canine and non-human primate (NHP) retinas. PSC axoneme was visualized by acetylated α-tubulin (AcTub) immunolabeling (magenta). N-hydroxysuccinimide (NHS) -ester counterstaining (gray) was used as a pan-protein morphological reference to visualize surrounding photoreceptor structures. All images are shown as maximum intensity projections. Scale bars, 1 μm, shown without correction for the expansion factor. All microscopy images in this figure are U-ExM images. **(B)** Schematic diagram illustrating basal body (BB) width measurement. **(C)** Cross-species comparison of BB width between rods and cones. Central lines indicate the mean, and error bars represent ± SD. No significant differences were observed among any pairwise comparisons (Welch's one-way ANOVA followed by Dunnett's T3 multiple-comparison test; n.s., $P > 0.05$).

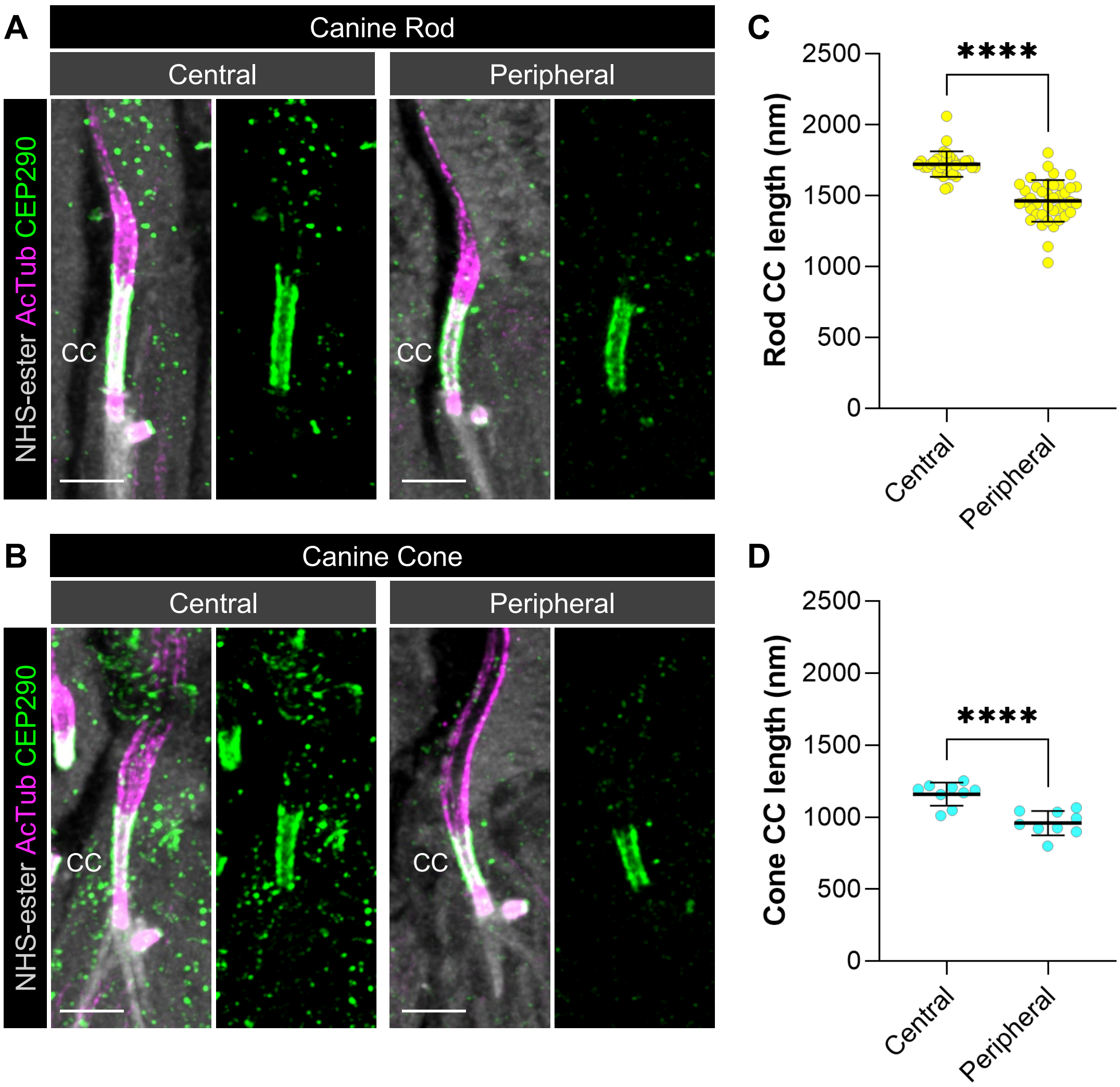

Fig. S2. Comparison of CC length between central and peripheral regions in the canine retina.

(A, B) Confocal images showing the length of the connecting cilium (CC) in the central and peripheral regions of the canine retina for rods (A) and cones (B). The ciliary axoneme and the CC were visualized by AcTub/CEP290 immunolabeling (magenta/green). NHS-ester counterstaining (gray) was used as a pan-protein morphological reference to visualize surrounding photoreceptor structures. All images are shown as maximum intensity projections. Scale bars, 1 µm, corrected for the expansion factor. All microscopy images in this figure are U-ExM images. **(C, D)** Quantification of CC length in the central and peripheral retina for rods (C) and cones (D). Central lines indicate the mean, and error bars represent ± SD. ****$P < 0.0001$, as assessed by Welch's *t*-test.

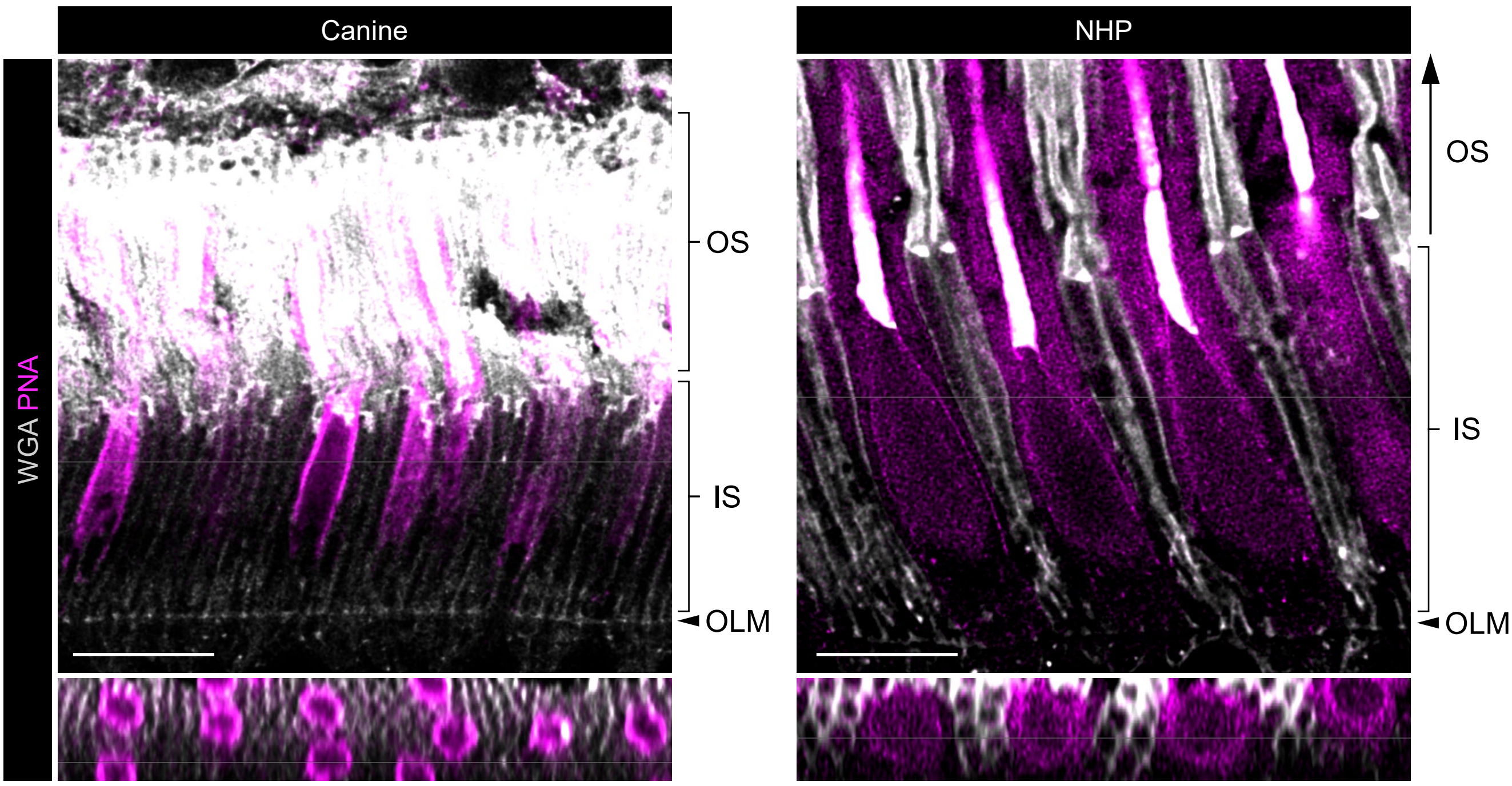

Fig. S3. Non-expanded retinal sections showing photoreceptor IS/OS morphology in canine and NHP retinas.

Representative single z-slice confocal images of non-expanded canine and NHP retinal sections stained with wheat germ agglutinin (WGA; gray) and peanut agglutinin (PNA; magenta). The field of view includes the outer limiting membrane (OLM), inner segment (IS), and outer segment (OS) regions, corresponding to the region shown in Fig. 1B. Lower panels show transverse views through the IS region. These images show that NHP photoreceptors have longer IS/OS profiles and greater transverse width at the IS level than canine photoreceptors. Scale bars, 10 µm.

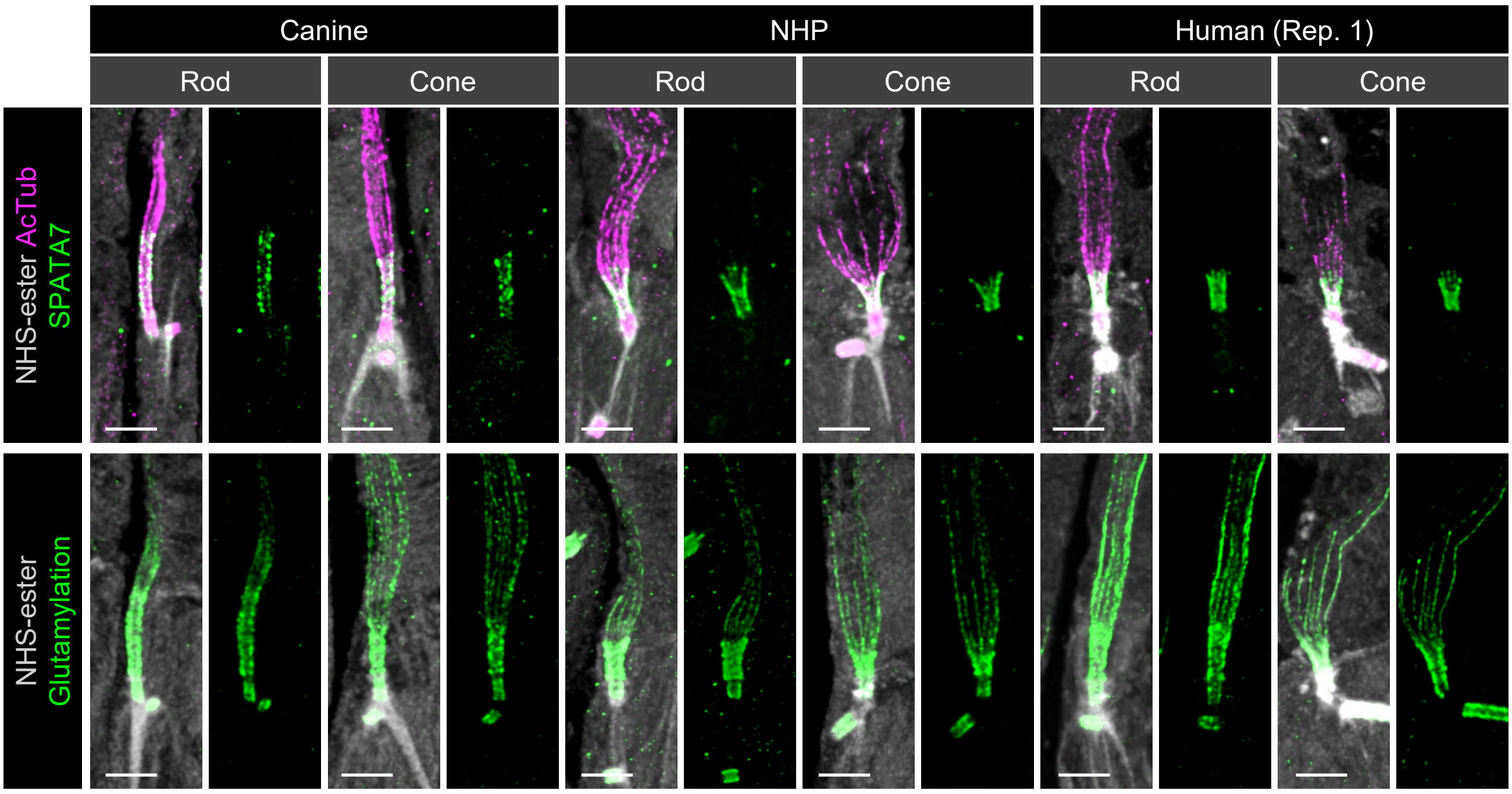

Fig. S4. Representative molecular markers of the CC.

Representative confocal images showing additional molecular markers of the microtubule-based structural core and CC length in rod and cone photoreceptors from canine, NHP, and human retinas. Upper panels show AcTub (magenta) and SPATA7 (green) immunolabeling, whereas lower panels show glutamylation (green) labeling of the PSC. Consistent with the results shown in Figs. 1 and 3, NHP and human photoreceptors have shorter CCs than canine photoreceptors, whereas the difference in CC length between rods and cones is maintained across all species. In addition, NHP and human cone photoreceptors possess markedly longer DCs than those in their rod counterparts and in canine photoreceptors. All images are shown as maximum intensity projections. Scale bars, 1 μm, corrected for the expansion factor. All microscopy images in this figure are U-ExM images.

Journal of Cell Science • Supplementary information

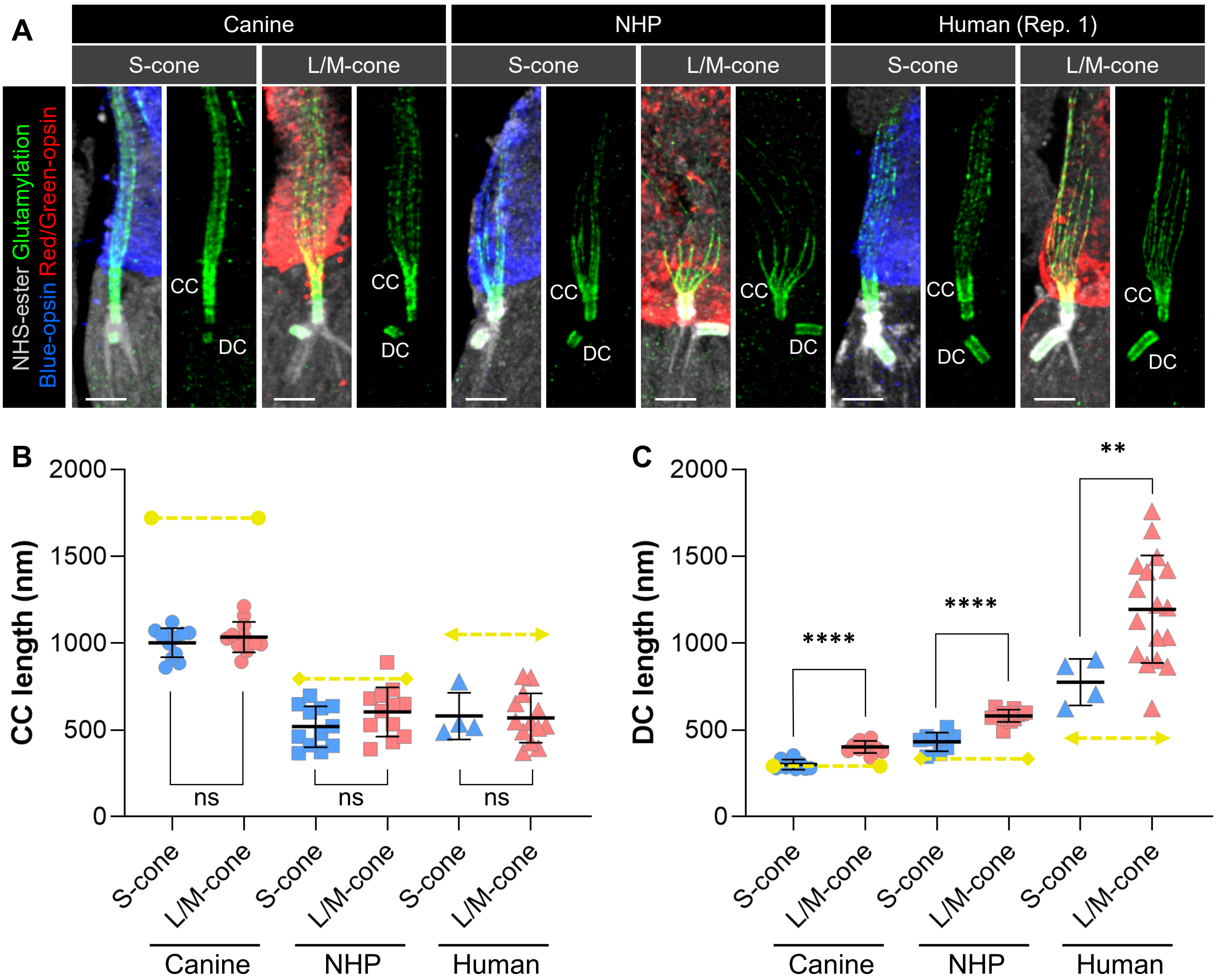

Fig. S5. Comparison of CC and DC length between S-cones and L/M-cones.

(A) Confocal images showing PSC structures in canine, NHP, and human retinas for S-cones (blue) and L/M-cones (red). The ciliary axoneme and CC were visualized by glutamylation immunolabeling (green). All images are shown as maximum intensity projections. Scale bars, 1 µm, corrected for the expansion factor. All microscopy images in this figure are U-ExM images. **(B, C)** Quantification of CC length (B) and DC length (C) in the three species (canine, circles; NHP, squares; human, triangles). Central lines indicate the mean, and error bars represent the ± SD. Yellow dashed lines indicate the mean values for rod photoreceptors of each species, derived from the corresponding measurements shown in Fig. 1E, J and Fig. 3C, E. Data were obtained from two or three individual eyes for each species. $^{**}P < 0.01$, $^{****}P < 0.0001$, as assessed by Welch's *t*-test.

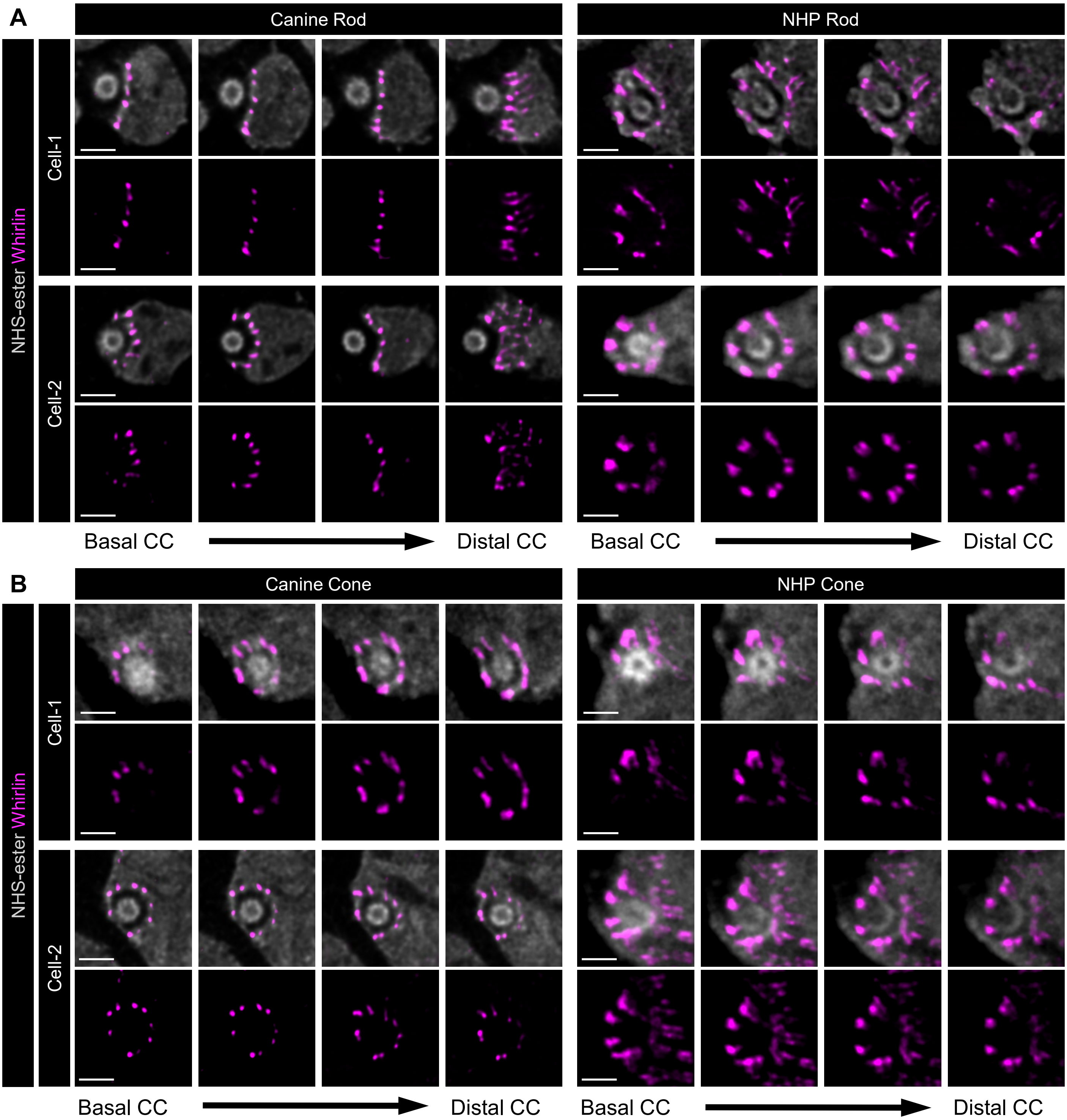

Fig. S6. Serial z-section analysis of Whirlin-positive periciliary structures in canine and NHP photoreceptors.

(A, B) Representative serial axial-view U-ExM images through the CC/periciliary region are shown for rods (A) and cones (B). Two representative photoreceptors are shown for each group: canine rods, NHP rods, canine cones, and NHP cones. In canine rods, Whirlin-positive structures were restricted to the IS-facing side of the CC, whereas in NHP rods, canine cones, and NHP cones, Whirlin-positive structures were distributed circumferentially around the CC. Many individual Whirlin-positive elements could be resolved as adjacent paired pillar-like structures in at least some z-sections, although this paired appearance was not evident for every element in every optical section. Scale bars, 500 nm, corrected for the expansion factor. All microscopy images in this figure are U-ExM images.

Journal of Cell Science • Supplementary information

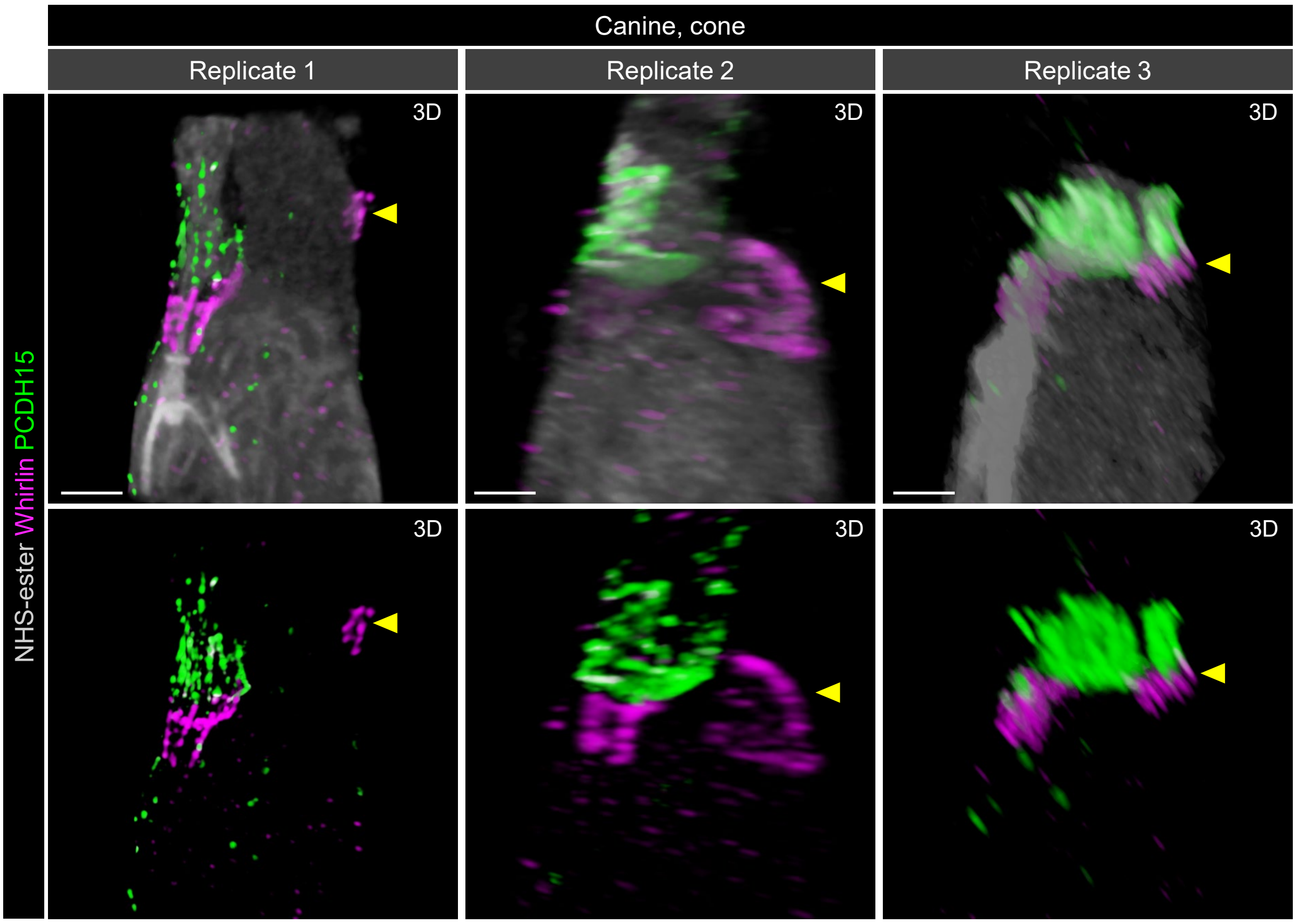

Fig. S7. Ectopic localization of Whirlin in canine cone photoreceptors.

Representative confocal images showing the localization of whirlin (magenta) and PCDH15 (green) in periciliary structures of canine cone photoreceptors from three independent biological replicates. Upper panels show merged images of whirlin, PCDH15, and NHS-ester counterstaining (gray), whereas lower panels show whirlin and PCDH15 labeling only. Yellow arrowheads indicate ectopic localization of whirlin. All images are shown as 3D-rendered images. Scale bars, 1 µm, corrected for the expansion factor. All microscopy images in this figure are U-ExM images.

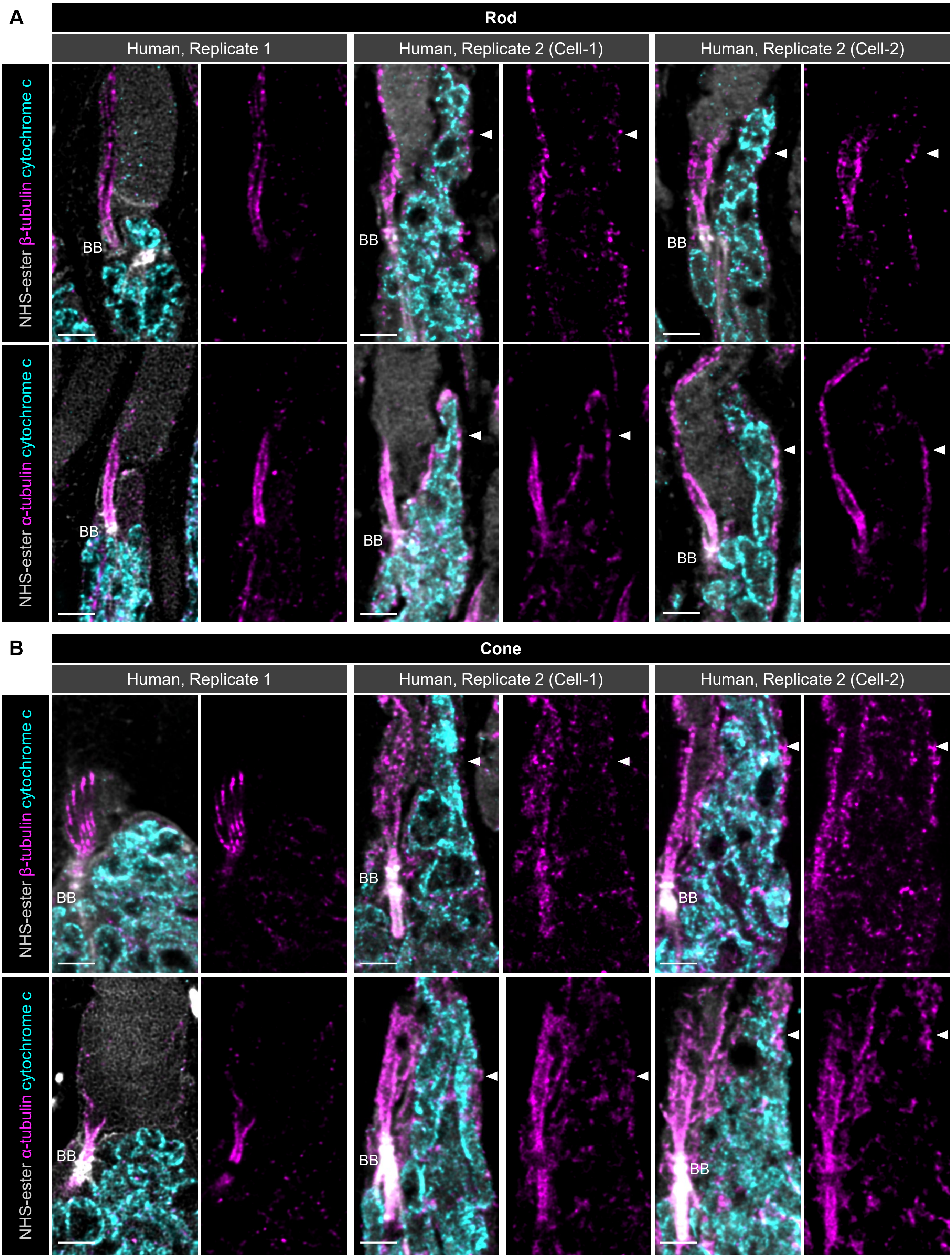

Journal of Cell Science • Supplementary information

Fig. S8. Tubulin organization in human accessory inner segment-like structures.

(A, B) Representative single optical section images of human rod (A) and cone (B) photoreceptors showing the distribution of IS mitochondria visualized by cytochrome c immunolabeling (cyan) and the microtubule cytoskeleton visualized by β-tubulin (upper panels) or α-tubulin (lower panels) immunolabeling (magenta). In human replicate 1, aIS-like structures and associated tubulin-positive extensions were not observed in either rods or cones. In human replicate 2, several rods and cones showed mitochondria-containing aIS-like structures extending alongside the OS. Both β-tubulin- and α-tubulin-positive signals were detected within these aIS-like regions, but the signals appeared variable and uneven, resembling the broader IS microtubule network rather than a scaffold unique to the aIS-like structure. One representative cell is shown for each staining condition and rods and cones in human replicate 1, and two representative cells are shown for each staining condition and rods and cones in human replicate 2. All images are shown as single optical sections. Scale bars, 1 µm, corrected for the expansion factor. All microscopy images in this supplementary figure are U-ExM images.

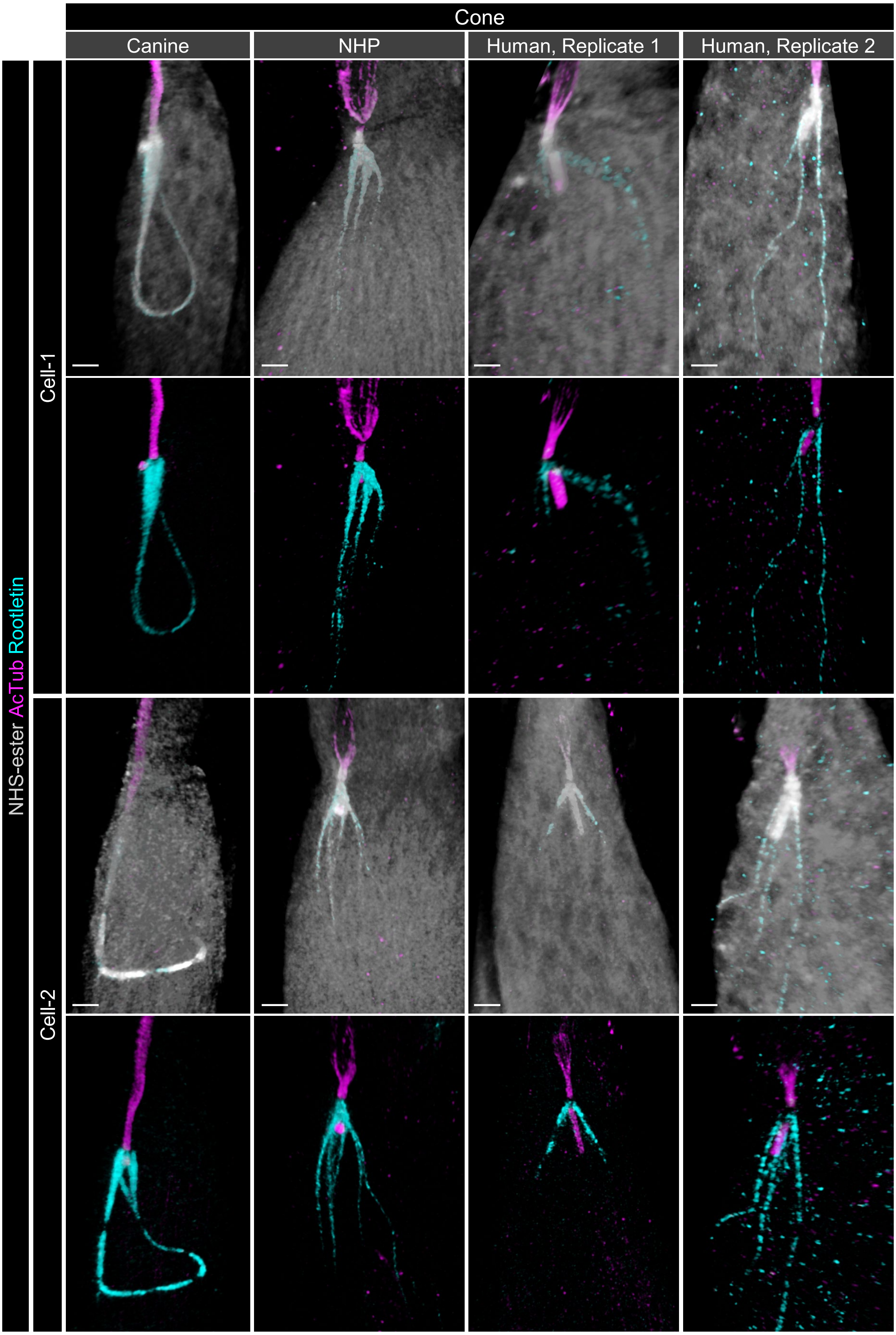

Journal of Cell Science • Supplementary information

Fig. S9. Additional examples of cone ciliary rootlet organization across species.

Representative U-ExM images of cone photoreceptors immunolabeled for rootletin, showing additional examples not included in Fig. 8B. Two cone photoreceptors are shown for each group: canine, NHP, human replicate 1, and human replicate 2. In canine cones, the terminal portions of the ciliary rootlet form a continuous arc-like configuration. In NHP and human cones, the rootlet termini are separated into thin fibers extending toward the basal IS region. Scale bars, 1 μm, corrected for the expansion factor. All microscopy images in this figure are U-ExM images.

Journal of Cell Science • Supplementary information

Table S1. List of archival retinas used in this study.

Species	ID	Sex	Age	Eye	Postmortem interval / Fixation conditions	Ref.
Canine	RC702	M	24 weeks	OS	< 5 mins / 4% FA 3 hrs, 2% FA 24 hrs	Takahashi et al., 2026
	RC703	M	24 weeks	OS		
	RC706	F	24 weeks	OS		
NHP (cynomolgus macaques)	21946	F	4 years	OD	< 20 mins / 4% FA 3 hrs, 2% FA 24 hrs	Jacobson et al., 2007
	21961	F	4 years	OS		
Human	125546 ODWE (replicate 1)	M	83 years	OS, OD	~ 3 hrs / 4% FA 7 days	NA
	123991 ODWE (replicate 2)	F	84 years	OS	~ 12 hrs / 4% FA 24 hrs	NA

F, female; FA, formaldehyde; M, male; NHP, non-human primate; OD, right eye; OS, left eye.

Table S2. Reagents and materials used for tissue processing and U-ExM.

Product	Supplier	Cat#
**Tissue processing and storage**		
20% paraformaldehyde solution	Electron microscopy sciences	15713-S
Sucrose	Fiser chemical	S2-500
Tissue-Tek O.C.T. Compound	Sakura finetek	4583
Tissue Embedding Disposable Molds	EBSciences	H1513
**U-ExM**		
14-mm microwell/35-mm petri dish	MatTek	P35G-1.5-14-C
12 mm Circular Cover Glasses	Fisher Scientific	12541001
Acrylamide (AA)	Sigma-Aldrich	A4058
Ammonium Persulfate (APS)	Bio-Rad	1610700
Formaldehyde solution	Sigma-Aldrich	F8775
ImmEdge Pen	Vector laboratories	H-4000
Nuclease-Free Water	Invitrogen	AM9937
N, N′-methylenbisacrylamide (BIS)	Sigma-Aldrich	M1533
Phosphate Buffered Saline (PBS), 10x	Bio-Rad	161-0780
Poly-D-Lysine	Gibco	A3890401
Sodium Acrylate (SA)	Sigma-Aldrich	408220
Sodium Chloride (NaCl)	Fisher Chemical	S271-3
Sodium Dodecyl Sulfate (SDS)	Fisher Chemical	BP166-500
Tetramethylethylenediamine (TEMED)	Bio-Rad	161-0800
Tris Base	Fisher Chemical	BP152-5

Journal of Cell Science • Supplementary information

Table S3. Antibodies and regents used for immunolabeling.

Primary antibodies

Antibody	Host Organism	Clonality / Isotype	Cat# or Ref.	Dilution	RRID
Acetylated α-tubulin	Mouse	Monoclonal IgG_{2b}	T7451	1/1000	AB_609894
Acetylated α-tubulin	Rabbit	Monoclonal IgG	ab179484	1/1000	AB_2890906
α-tubulin	Rabbit	Monoclonal IgG	ab18251	1/500	AB_2210057
β-actin	Rabbit	Polyclonal IgG	ab8227	1/200	AB_2305186
Blue opsin	Rabbit	Polyclonal IgG	AB5407	1/500	AB_177457
β-tubulin	Mouse	Monoclonal IgG_1	T4026	1/500	AB_477577
CEP164	Rabbit	Polyclonal IgG	22227-1-AP	1/200	AB_2651175
CEP290	Rabbit	Polyclonal IgG	22490-1-AP	1/200	AB_10973679
cytochrome c	Sheep	Polyclonal IgG	C9616	1:200	AB_532232
Glutamylation (GT335)	Mouse	Monoclonal IgG_1	AG-20B-0020	1/1000	AB_2490210
LCA5 (lebercilin)	Rabbit	Polyclonal IgG	19333-1-AP	1/200	AB_2878576
PCDH15	Sheep	Polyclonal IgG	AF6729	1/300	AB_10892338
POC5	Rabbit	Polyclonal IgG	A303-341A	1/200	AB_10971172
Red/Green opsin	Rabbit	Polyclonal IgG	AB5405	1:300	AB_177456
Rhodopsin	Rabbit	Polyclonal IgG	AB9279	1/1000	AB_11210489
Rootletin	Human	Monoclonal IgG	HCA009	1/200	AB_2085504
Rootletin	Mouse	Monoclonal IgG_1	sc-374056	1/200	AB_10918081
RP1	Chicken	Polyclonal IgY	Custom (Liu et al., 2002)	1/300	NA
SPATA7	Rabbit	Polyclonal IgG	12020-1-AP	1/200	AB_2195380
Whirlin	Rabbit	Polyclonal IgG	25881-1-AP	1/200	AB_2880280

Secondary antibodies

Target	Host Organism	Clonality / Isotype	Fluorescent	Cat#	Dilution	RRID
Chicken IgY (H+L)	Goat	Polyclonal IgG	Alexa Fluor 488	A11039	1:1000	AB_2534096
Mouse IgG_1	Goat	Polyclonal IgG	Alexa Fluor 488	A21121	1:1000	AB_2535764
Mouse IgG_{2b}	Goat	Polyclonal IgG	Alexa Fluor 488	A21141	1:1000	AB_2535778
Sheep IgG (H+L)	Donkey	Polyclonal IgG	Alexa Fluor 488	A11015	1:1000	AB_2534082
Human IgG (H+L)	Goat	Polyclonal IgG	Alexa Fluor 568	A21090	1:1000	AB_2535746
Mouse IgG_1	Goat	Polyclonal IgG	Alexa Fluor 568	A21124	1:1000	AB_2535766
Mouse IgG (H+L)	Donkey	Polyclonal IgG	Alexa Fluor 568	A10037	1:1000	AB_11180865
Rabbit IgG (H+L)	Goat	Polyclonal IgG	Alexa Fluor 568	A11036	1:1000	AB_10563566
Rabbit IgG (H+L)	Donkey	Polyclonal IgG	Alexa Fluor 568	A10042	1:1000	AB_2534017

Fluorescent conjugates

Product	Fluorescent	Supplier	Cat#	Stock solution	Dilution
Hoechst 33342	Hoechst 33342	Invitrogen	62249	20 mM	1:5000
NHS-ester	Atto 647N	Sigma-Aldrich	18373	2 mg/mL	10 µg/mL
PNA	Alexa Fluor 568	Invitrogen	L32458	1 mg/mL	10 µg/mL
WGA	Alexa Fluor 488	Invitrogen	W11261	1 mg/mL	10 µg/mL

RRID, research resource identifiers.

Table S4. Source data for all graphs and quantitative analyses

Available for download at

https://journals.biologists.com/jcs/article-lookup/doi/10.1242/jcs.265080#supplementary-data

Journal of Cell Science • Supplementary information
