# Supplementary material for: Cross-species molecular mapping of the photoreceptor sensory cilium and periciliary structures identifies conserved and species-specific architectural features
Source: J Cell Sci. 2026 Oct 22;139(21):jcs265080. doi: 10.1242/jcs.265080 (PMC13723404; doi:10.1242/jcs.265080)
Supplement: Supplementary file 2 [file joces-139-265080_review_history.pdf]

Journal of Cell Science

Cross-species molecular mapping of the photoreceptor sensory cilium and periciliary structures identifies conserved and species-specific architectural features

Kei Takahashi, Mayuna Obayashi, Raghavi Sudharsan and William A. Beltran
DOI: 10.1242/jcs.265080

Editor: Guillaume Jacquemet

Review timeline

Original submission:	27 May 2026
Editorial decision:	15 June 2026
First revision received:	4 August 2026
Editorial decision:	18 August 2026
Second revision received:	18 August 2026
Accepted:	19 August 2026

Original submission

First decision letter

MS ID#: jcs.265080

MS TITLE: Cross-species molecular mapping of the photoreceptor sensory cilium and periciliary complexes identifies conserved and species-specific architectural features

AUTHORS: Kei Takahashi; Mayuna Obayashi; Raghavi Sudharsan; William A. Beltran

ARTICLE TYPE: Research Article

Dear Dr Beltran,

We have now reached a decision on the above manuscript.

As you will see, the reviewers raise a number of substantial criticisms that prevent me from accepting the paper at this stage. They suggest, however, that a revised version might prove acceptable, if you can address their concerns. If you think that you can deal satisfactorily with the criticisms on revision, I would be pleased to see a revised manuscript. We would then return it to the reviewers.

Reviewer 1

SUMMARY OF THE ADVANCE MADE IN THIS PAPER AND ITS POTENTIAL SIGNIFICANCE TO THE FIELD

The study by Takahashi et al. provides a comparative analysis of photoreceptor sensory cilia organization across canine, non-human primate, and human retinas. The authors utilize expansion microscopy, which enables visualization of nanoscale structural features and reveals both conserved and species-specific specializations of rods and cones. This work provides additional detail regarding the microtubule-based architecture of the connecting cilium and periciliary complex in large mammals. However, the study is largely descriptive and offers limited functional or mechanistic insight into the biological significance of the structural differences observed. Furthermore, it is unclear whether measurements of the dimensions of specific subcellular

© 2026. Published by The Company of Biologists under the terms of the Creative Commons Attribution License (https://creativecommons.org/licenses/by/4.0/).

compartments using expansion microscopy provide any advantage over corresponding measurements obtained from conventional, non-expanded EM images. Expansion microscopy may also introduce artifacts due to uneven expansion of different cellular structures. Several interpretations appear overstated or insufficiently supported by the presented images. Overall, the data presented in this manuscript are largely confirmatory and therefore unlikely to be of broad interest to a general audience of cell biologists.

SUGGESTIONS TO AUTHORS

1. The statement "Although the tubulin-based architecture of the PSC has not been systematically compared across species, previous work has shown that periciliary structures already exhibit pronounced interspecies and subtype-dependent variation: calyceal processes are well developed in NHP and human photoreceptors but absent or vestigial in rodents" is misleading. Calyceal processes are actin-based structures, not tubulin-based structures. Please rephrase to clearly distinguish between the tubulin-based ciliary scaffold and associated actin-based periciliary extensions.
2. The statement that "Overall, the NHP retina exhibits larger photoreceptor inner segments (IS) and outer segments (OS) than the canine retina" requires clarification. It is unclear whether "larger" refers to photoreceptor length or diameter. In addition, the cited references do not directly support claims regarding photoreceptor cell size; rather, they discuss differences in overall retinal size and photoreceptor density, not photoreceptor cell size. Although NHP cones have been shown to be larger than those in rodents, the size of NHP rods appears comparable to that in rodents. Therefore, this interpretation should be supported by images from non-expanded samples.
3. The sentence "…the overall dimensions of the tubulin-based PSC scaffold appeared broadly similar between canine and NHP photoreceptors (Fig. 1C)" is difficult to interpret. First, the term "tubulin-based PSC scaffold" is not clearly defined. Second, Figure 1C does not show quantitative measurements supporting this conclusion. The authors should either define the structural feature being referenced and provide the corresponding quantification or revise the statement.
4. Figure 2E. The cartoon indicates "Apical end of the CC" at position 0, whereas the legend states that "The distal end of the CC was used to define the 0 nm position." These descriptions should be made consistent. In addition, the term "apical region of PSCs" is not properly defined. Logically, the apical region of the PSC should correspond to the outer segment tip.
5. Figure 2F legend. The statement "The CC and axoneme were visualized by AcTub/SPATA7 immunolabeling" does not explain that SPATA7 labels the connecting cilium region. Please clarify which structure is labeled by each marker.
6. Figure 3G presents cartoons summarizing bulge-region features in canine and NHP rods and cones. Please move this panel to Figure 2, as Figure 3 which focuses exclusively on human data.
7. Figure 4D. The claim that in NHP rods the whirlin-positive structures are arranged as "pairs of adjacent pillar-like structures around the CC" is not supported by the presented images. Some of the whirlin structures in the NHP cells appear to be non-paired. The authors should either provide stronger visual support for this interpretation or modify the description.
8. The authors claim that calyceal processes are rudimentary or absent in canine rods, in contrast to NHP rods. However, the PCDH15 and actin staining in Figure 5D appears similar in canine and NHP rods. This interpretation should therefore be reconsidered.
9. Figure 6A. The text states: "Actin filaments extending toward these PCDH15-positive structures were also evident, further supporting a NHP-like periciliary organization in human photoreceptors (Fig. 6A)." However, panel A does not include actin staining.
10. I have a significant concern about the quality of tissue preservation and/or processing shown in Figure 7A. Why are mitochondria not stained in two out of the three cells shown in panel "human replicate 1"? And why is cytochrome C brightly stained in one of the canine outer segments? These data do not meet the standard of publication quality. Also, the authors state that no additional tubulin-based scaffold was detected on the side of the IS opposite the ciliary axoneme, in contrast to a prior EM study. However, tubulin staining is clearly present within the accessory inner segment in Human replicate #2. The authors should address and reconcile this inconsistency.

Reviewer 2

SUMMARY OF THE ADVANCE MADE IN THIS PAPER AND ITS POTENTIAL SIGNIFICANCE TO THE FIELD

© 2026. Published by The Company of Biologists under the terms of the Creative Commons Attribution License (https://creativecommons.org/licenses/by/4.0/).

The new expansion microscopy manuscript, "Cross-species molecular mapping of the photoreceptor sensory cilium and periciliary complexes identifies conserved and species-specific architectural features," by Takhashi et al., provides an important comparison of canine, non-human primate (NHP), and human photoreceptor cilia that highlights new cross-species differences among these elaborate cells. The manuscript reports important findings that should be published after improved quantitative analysis, as well as careful revisions and clarifications. The advances made in the paper include:

1. The connecting cilium (CC) proteins CEP290 and SPATA7, as well as the enrichment of glutamylated microtubules in the CC, are all shorter in NHP rods and cones compared to canine counterparts. Although the manuscript provides a comprehensive comparative analysis, there are unexplained CC structural differences based on the staining experiments performed (see below).

2. NHP and human cone daughter centrioles are elongated compared to other measured photoreceptors, in line with other recent findings.

3. The "bulge" of the OS axoneme immediately above the CC appears more pronounced in cones of both species.

4. Whirlin localization in NHP rods, as an indicator of the subcellular organization of the periciliary protein complexes, was significantly different than canine rods, which exemplifies a major species difference. Missing quantitative data is requested in a revision in order to support this important finding.

5. An ExM evaluation of the human accessory inner segment in human photoreceptors was performed.

The data are significant since the study includes novel U-ExM data using NHP and human retinal tissue. The findings in the paper provide critical cross-species information needed to understand the differences in mammalian rods in order to interpret findings among different studies with different models. The human photoreceptor data is an essential inclusion. The U-ExM data are of excellent quality and value. However, a limitation to the study is that the conclusions are strictly based on U-ExM data. While ExM is a robust technique for assessing ciliary structural differences in photoreceptors, a lack of complementary methods prevents a complete characterization of the described cross-species structural differences.

SUGGESTIONS TO AUTHORS

Major comments for the revision

Needed are discussions of the possible functional significance of longer daughter centrioles in NHP cones, the shortening of CCs in NHPs compared to canines, and the unique whirlin distribution in NHP rods.

Overall the abstract is esoteric and requires knowledge of cilia and photoreceptor ultrastructure. Incorrect or confusing terminology is used that should be clarified. The abstract should also describe the key structural and functional importance of the connecting cilium and periciliary region and describe their relevance to retinal disease.

Fig. 1E and Fig. 2A/B depict results from similar analyses, yet the measurements and some of the comparisons draw different conclusions. For example, canine cone CC length is (presumably) not significantly different from NHP rod CC length in Fig. 2C. These differences in the data should be explored and discussed. Ideally, the datasets should be presented together to assist interpretation. Furthermore, the same canine measurements were performed in Takahashi et al. (2025). Yet, there seems to be some variability in the measurements from Fig. 3H, 5K, and 6S of the 2025 paper compared to Fig. 1E of this manuscript. Please indicate that the same differences were replicated/confirmed between studies.

Â·Line 178: The conclusion that human rod and cone CCs were shorter than canine and "more closely resemble" NHP CCs is vague. Why aren't all the human vs. NHP vs. canine measurement data

© 2026. Published by The Company of Biologists under the terms of the Creative Commons Attribution License (https://creativecommons.org/licenses/by/4.0/).

directly compared? These are the truly novel comparisons in this work. For this reason, the data in Supplementary Figure 4A should be presented in the main figure.

Line 220: There are six whirlin puncta in the example in Fig. 4B, but are there always six? The rod in Fig. 4C may have more than six. This localization should be quantified. Please provide the full Fig. 4A-C dataset of images, from which this important conclusion is derived.
·(related) Fig. 5B,C: The pillars, again, should be quantified and compared. It would be specifically interesting to compare them between canine cones and NHP rods.
·(related) Line 257: The conclusion of five pairs vs. six pairs is potentially of notable, functional significance but cannot be broadly made without more examples or, preferably, quantification with statistics.

Fig. 7B: It is unclear from these cone examples if the human cones have aIS-like extensions. The top human replicate 2 cone shows mitochondrial staining in this region above the BB, but it is not evidently adjacent to the OS, and so this could represent an extension of the IS proper. In the second human replicate 2 cone, the indicated region looks like the periciliary regions highlighted with Fig. 6. Thus, the aIS distinction requires mitochondrial labeling and clear apposition to the OS from the same cell (as shown for rods in Fig. 7A). Can such an example be provided for cones?

Line 337/Fig. 8B: There are no apparent ciliary rootlet differences among the different cone examples presented here. Each of the examples could be considered branched and curvilinear. Please provide more examples or measurements. Otherwise, the strong conclusions about rootlet differences should be removed from the text.

Detailed comments

Abstract

Line 30, first sentence: This is a confusing start: Photoreceptor sensory cilia (PSC) contain the OS, along with the BB, CC, and axonemes within, and so it should not be written that the PSC supports outer segment formation; rather, the PSC includes the structures that support the proper formation (i.e., the axoneme and BB centrioles) and organization of outer segment discs. The "PSC" terminology, while consistent with the author group's past literature, should be reconsidered in some cases or at least explained for the sake of new readers not familiar with the past papers, such as experts in other types of cilia.

Line 31, still the first sentence: The term "subtype-dependent organization" is also confusing without any additional context. This terminology is used again on line 34, and it is used throughout the group's papers. I would suggest orienting readers to indicate the meaning of subtype (as in Takashi et al. 2025), which, in the context of this manuscript, refers to the major photoreceptor cell type, more specifically, rods vs. cones. On, for example, lines 157 and 163 (but also elsewhere in the manuscript), the authors use the term "rod-cone differences," which is preferable, clearer, and better aligned with the literature. Such terminology vagaries are potentially exacerbated by suggestions from a writing tool that is incompletely trained on the appropriate literature. In general, more cautious evaluation of the terms and wording provided from such tools may be needed.

Line 36: The "bulge region" is also confusing without any context or prior knowledge (see below for more about this term).

Line 44: "Principles" is another confusing term when describing cell structures. It could be replaced with something more clear, like "structural features."

Introduction

Lines 50-53: The "tubulin"/microtubule core should be distinguished as the axoneme in this section since this is the prevalent terminology. The CC has an axoneme that continues into the OS, and so the axoneme is part of both compartments.

© 2026. Published by The Company of Biologists under the terms of the Creative Commons Attribution License (https://creativecommons.org/licenses/by/4.0/).

Line 53: The "bulge region," defined as such in Mercey et al. (2022), should be characterized with a description. While "bulge region" may serve as a technical term for the structure, it should be redefined in this manuscript for the unfamiliar. Others may understand it as the portion of the axoneme in the nascent disk region/OS base/proximal OS.

Line 57: The writing here suggests that the periciliary region surrounds the apical inner segment. I think the meaning is that that periciliary region is located at the apical IS or is part of it.

Line 58: The ciliary pocket resembles such a structure in primary cilia, but the periciliary region does not (it is unique to photoreceptors). As a note: the periciliary membrane complex (PMC) abbreviation, which is familiar to some, could also create some unintended confusion with the "PSC" abbreviation.

Line 62: The USH proteins and Usher syndrome (or any disease relevance of the work) are not properly introduced. Also, please indicate which species and cell types have the described USH protein distributions.

Line 66: Here it is indicated that there are multiple periciliary complexes.

Line 67: This final sentence of the paragraph is misleading. In fact, dissecting the rod-cone and species differences in the cilia is a hot topic in the literature, and there is already published detailed knowledge, including among the cited references (Takahashi et al. 2025; Sahly et al. 2012; Verschueren et al. 2022; Lewis et al. 2025; etc.).

Results

Line 91: The title of the section is confusing. How is the PSC architecture conserved if there are species-specific differences?

Line 94: More terminology uncertainty: the standard meaning of "basal body" in the field is mixed. In Gilliam et al. (2012), both centrioles were termed "BBs." Whereas in Wensel et al. (2021) and Mercey et al. (2022), they are distinguished as the mother centriole "MC" and "DC." Please provide a description of the BB region as the region comprising the MC, the DC, and the surrounding area that contains pericentriolar satellites, and then specify that the MC is interchangeably labeled as the "BB" in this manuscript.

Line 101: The expansion factor calculations, although based on a published method, seem to be error-prone. The range of measured values in Supplementary Figure 1C is 0.6 - 1 micron. This is a potential cell-to-cell EF variation of 2.6 - 4.3. Could the authors comment on the feasibility of optimization in which the EF is calculated individually for each cell by, for example, measuring the MC/BB width from each cilium for normalization to the standard? This seems to be important for accuracy, considering the aforementioned cell-to-cell variation and possibility of ongoing expansion during the imaging period. Related, Line 512: Please provide additional details of the EF calculation. Were FWHM intensity values from line scans used to calculate the MC/BB widths?

Fig. 1C: Please remark on the CEP290 labeling in the "bulge region" of NHP rods. Is this high background or real labeling? Also, please remark on the distance of the DC from the MC throughout the data. There appears to be wide variability; the published centriol-centriole distance from mouse rod cryoET data in Gilliam et al. (2012) is ~500 +/- 100 nm. In some U-ExM examples throughout these figures the MC-DC distance noticeably exceeds that distance.

Line 130, Fig. 1 F: Please confirm in the text or figure legend that CC axoneme width measurements were calculated from the tubulin immunolabeling (anti-acTub).

Line 142: A brief description of the RP1 and LCA5 proteins should be provided.

Line 149: Terminology consistency is needed: the "distal PSC" likely means the OS or the OS axoneme.

© 2026. Published by The Company of Biologists under the terms of the Creative Commons Attribution License (https://creativecommons.org/licenses/by/4.0/).

Figs. 2E-G have less value since they have no quantification associated with the images. Additionally, the NHS-ester stained OSs in Fig. 2F are distended and difficult to interpret. These images should be considered as supplemental views to the more informative longitudinal images already presented.

Line 157: The authors must qualify that the larger OS axoneme bulge difference conclusion finding is qualitative or more concisely state that the OS axoneme bulge (widening) was more pronounced based strictly on morphological observations.

Line 169: Noticeable morphological variation is suggested in the human imaging data of Fig. 3A, but this is not apparent and should be discussed. Better yet, please provide lower magnification views for the assessment of the overall tissue quality.

Line 181: The authors should reference the Moye et al. (2026) Am J Hum Genet paper (PMID: 41742423), which shows a particularly striking elongated daughter centriole in an expanded human cone (Fig. 3C). Many readers will be interested in comparing the data.

Line 192: Please qualify these "bulge" region observations as just that: morphological observations. Also, the "bulge" in the axoneme appears "wider" or more "distended." The term "extended" is not as clear, and the suggestion of "greater expansion" is potentially confusing for obvious reasons.

Fig. 3G should be removed due to the lack of quantification proving the OS axoneme is wider in cones. The morphological conclusion is apparent (and valid) based on the images themselves.

Line 205: Curiously, the region that is called the IS/OS boundary here is termed the IS/OS junction in the referenced Sahly et al. 2012 paper. Why the distinction?

Line 207: Please provide an appropriate reference for this statement about rod calyceal processes.

Line 213: It seems like an overstatement that NHS-ester counterstaining provides "clear delineation of periciliary subcompartments." Perhaps it "helps" determine the subcompartment locations?

Line 226: What are "singlets"? Are they branches from the "pillars"? The conclusion of the line scans in Fig. 4D needs more explanation. Are these same patterns found in other rods?

Line 236: Based on the examples in Fig. 4F, the actin staining seems comparable between canine and NHP rods. Therefore, please reconsider terms like "prominent" and "robust." The morphological observation of the alignment of the actin streaks with the NHP PCDH15 is accurate.

Line 242: "Weakly developed" is a vague term. What is the meaning? Do canine rods have calyceal processes? Perhaps they are smaller and more difficult to capture with immunofluorescence?

Line 255: Here, the "pillar-like" elements are called "paired doublet-like" elements. Much more consistency and better clarity about these novel structures are needed.

Line 277: Clarification needed: Only 1 rod in Fig. 6A shows calyceal-like process PCDH15 labeling.

Line 293: "Highly recent" is a vague term. What is the meaning?

Line 301: Please clarify: Do all the rods in the human donor replicate 1 retina show aIS-like structures?

Line 307: No tubulin scaffold is reported in the aIS-like structure; however, there appears to be beta-tubulin staining in human replicate 2 in Fig. 7A (actually right where the arrowhead is pointing). Please show the beta-tubulin channel only. What does this staining represent?

Line 313: The term "abnormality" should be reconsidered.

Line 330: Considering that the rootlet is "tethered" to the BB (see Wensel et al. 2021 and SÃ¡nchez-Bellver et al. 2021), it more logically extends down from the BB into the IS cytoplasm.

© 2026. Published by The Company of Biologists under the terms of the Creative Commons Attribution License (https://creativecommons.org/licenses/by/4.0/).

Line 335: Without rootlet measurements, please qualify the conclusions as "apparent," "appeared," or based on "morphological observation."

Discussion

Line 361: This sentence is redundant and difficult to read. Consider removing it to get to the next point.

Line 372: "Comprehensive inclusion" reads like flowery jargon that should be reconsidered.

Line 405: Is an EM paper being referenced here, or is this referencing unpublished observations?

As detailed above, the functional implications of the major findings in this paper should be included in the manuscript.

Materials and Methods

Line 496: For the sake of readers unfamiliar with the ExM technique, please provide more details for "post-expansion immunohistochemistry, counterstaining, and second-round expansion." Also, how were gels mounted for imaging? How long were the imaging periods? Expansion potentially continues in a water-based mountant during long imaging periods. Could this be an important technical consideration with this method?

Figue Legends

Line 639, Figure 1 Legend (and throughout): Please confirm that all imaging data are U-ExM.

Line 644, MIP as an abbreviation for maximum intensity projection, is unnecessary and could be confused with its use as an abbreviation for microtubule inner protein by cilia/flagella experts

Line 679: Describing NHS-ester staining of the "photorecepotor soma" is unusual. The staining is either OS membranes or the apical IS.

First revision

Author response to reviewers' comments

We would like to thank the reviewers for their valuable feedback and appreciate their insightful comments and suggestions. We have addressed these comments and have detailed our responses in the point-by-point reply below. For clarity, we have highlighted the author's responses and the modifications in the main text in blue.

Reviewer #1

SUMMARY: The study by Takahashi et al. provides a comparative analysis of photoreceptor sensory cilia organization across canine, non-human primate, and human retinas. The authors utilize expansion microscopy, which enables visualization of nanoscale structural features and reveals both conserved and species-specific specializations of rods and cones. This work provides additional detail regarding the microtubule-based architecture of the connecting cilium and periciliary complex in large mammals. *However, the study is largely descriptive and offers limited functional or mechanistic insight into the biological significance of the structural differences observed. Furthermore, it is unclear whether measurements of the dimensions of specific subcellular compartments using expansion microscopy provide any advantage over corresponding measurements obtained from conventional, non-expanded EM images. Expansion microscopy may also introduce artifacts due to uneven expansion of different cellular structures. Several interpretations appear overstated or insufficiently supported by the presented images. Overall,*

© 2026. Published by The Company of Biologists under the terms of the Creative Commons Attribution License (https://creativecommons.org/licenses/by/4.0/).

the data presented in this manuscript are largely confirmatory and therefore unlikely to be of broad interest to a general audience of cell biologists.

Response: We thank the reviewer for the careful evaluation of our manuscript and for the detailed comments on terminology, figure presentation, and the distinction between quantitative conclusions and qualitative morphological interpretations. However, we respectfully but firmly disagree with several aspects of the reviewer's overall assessment, particularly the statements that the study is "largely descriptive," "largely confirmatory," provides "limited advantage over conventional EM", and is "unlikely to be of broad interest to cell biologists". We believe that these statements underestimate both the conceptual advance and the technical value of the study.

First, we disagree that the structural focus of this study limits its biological value. The primary objective of this work was to establish a cross-species structural framework for the photoreceptor sensory cilium and associated periciliary structures that resolves differences between rods and cones. Using U-ExM, we identified multiple nanoscale features and protein localization patterns that had not been systematically resolved by conventional approaches. We agree that the functional and mechanistic consequences of these architectural differences are important questions. However, these questions require additional experimental systems and functional assays and are therefore beyond the central scope of the present manuscript. The present study provides the comparative nanoscale molecular framework required for such future mechanistic studies to be designed and interpreted.

Second, we disagree that U-ExM provides limited advantage over conventional, non-expanded EM for this study. Conventional EM is indispensable for resolving ultrastructure and for precise nanoscale measurements. However, the main advantage of expansion microscopy here is not simply dimensional measurement, but relatively high-throughput, molecularly resolved imaging across multiple markers, photoreceptor subtypes, species, and tissue specimens. By linking nanoscale structural features with specific protein localization patterns, we were able to identify or redefine architectural features that were previously overlooked, incompletely characterized, or ambiguously interpreted. Because EM requires specialized sample preparation and is relatively low-throughput, EM-based studies are often limited to selected qualitative ultrastructural observations **(PMID: 20368623, 31843915, 39820780, 3987175)**. Immuno-EM can provide molecular information, but it is generally not well suited for systematic multi-marker comparisons across multiple species and between rods and cones. In contrast, U-ExM can be applied to conventional formaldehyde-fixed cryopreserved retinal samples and is compatible with standard fluorescence microscopy and immunolabeling tools. This versatility enabled the multi-marker, cross-species, and rod-cone- resolved comparative analysis presented in this manuscript.

Third, we agree that expansion-related artifacts are an important technical consideration, but we do not agree that this concern invalidates the major conclusions of the study. U-ExM was specifically developed to improve ultrastructural preservation and expansion isotropy compared with earlier expansion microscopy approaches, and its ability to preserve nanoscale cellular architecture has been demonstrated previously **(Gambarotto et al., 2019; PMID: 30559430)**. Importantly, U-ExM- derived measurements in photoreceptor sensory cilia are also broadly consistent with measurements obtained using independent ultrastructural methods. For example, Mercey et al. reported a CEP290- labeled connecting cilium length of ~1,500 nm in mature mouse rods using U-ExM **(Mercey et al., 2022; PMID: 35709082)**, whereas Gilliam et al. reported a ciliary membrane length of ~1,100 nm in mouse rods using cryo-electron tomography **(Gilliam et al., 2012; PMID: 23178122)**. Although these values are not completely identical, they are broadly comparable given the differences in measured structures and markers. This supports the reliability of U-ExM-derived dimensional measurements in the photoreceptor sensory cilium. We acknowledge that, as with any expansion-based method, potential distortion cannot be completely excluded when interpreting absolute dimensions. However, the main quantitative conclusions of the present study are based primarily on relative comparisons performed under the same U-ExM workflow, using the same expansion factor strategy, comparable imaging conditions, and consistent measurement criteria across species and between rods and cones. Therefore, while U-ExM-derived absolute values should be interpreted with appropriate caution, relative comparisons among U-ExM-derived measurements within this controlled experimental framework are, in our view, robust and suitable for the cross-species and rod-cone-resolved analyses presented here.

© 2026. Published by The Company of Biologists under the terms of the Creative Commons Attribution License (https://creativecommons.org/licenses/by/4.0/).

Fourth, we strongly disagree that the data are “largely confirmatory” or unlikely to be of broad interest to cell biologists. Although classic EM studies have provided important ultrastructural descriptions of photoreceptors, detailed molecular mapping and quantitative analysis of PSC architecture in NHP retina remain limited. Similarly, recent U-ExM studies have provided important but partial insights into human photoreceptor tubulin axoneme architecture **(PMID: 39528655, 40704549, 41742423)**; however, our study extends beyond these reports by providing a systematic, quantitative comparison across species and between rods and cones. In addition, our analysis of periciliary structures, particularly differences in the organization of Whirlin-positive periciliary membrane domains among species and between rods and cones, clarifies structural features that were difficult to define using previous approaches. Thus, the present work is not a confirmatory extension of prior observations, but provides a new comparative molecular framework for photoreceptor ciliary biology, evolutionary cell biology, organelle specialization, and the interpretation of animal models of human retinal disease. We also believe that the manuscript fits well within the scope of *Journal of Cell Science*, which explicitly includes topics such as “Cilia and flagella” and “Imaging” and has recently published multiple studies using expansion microscopy to investigate ciliary biology **(PMID: 40704549, 41064933, 41017282, 37013443)**. By applying U-ExM to retinal tissue and extending the analysis beyond the primary ciliary axoneme to associated periciliary and cytoskeletal structures, this study directly connects advanced imaging, ciliary biology, and organelle specialization, and should therefore be of clear interest to the JCS readership.

Comment 1: The statement "Although the tubulin-based architecture of the PSC has not been systematically compared across species, previous work has shown that periciliary structures already exhibit pronounced interspecies and subtype-dependent variation: calyceal processes are well developed in NHP and human photoreceptors but absent or vestigial in rodents" is misleading. Calyceal processes are actin-based structures, not tubulin-based structures. Please rephrase to clearly distinguish between the tubulin-based ciliary scaffold and associated actin-based periciliary extensions.

Response: We did not intend to imply that calyceal processes are tubulin-based structures. In the original sentence, we aimed to distinguish the tubulin-based PSC axonemal architecture from associated periciliary structures, including actin-based calyceal processes. Nevertheless, we agree that the wording could be read as conflating these two structural systems. We have therefore revised the sentence to clearly separate the microtubule-based PSC axoneme from actin-rich periciliary extensions.

Line 87: Although systematic cross-species comparison of the microtubule-based PSC axoneme remains limited, associated periciliary structures, including actin-rich calyceal processes, are already known to exhibit pronounced interspecies and rod-cone variation, with calyceal processes being well developed in NHP and human photoreceptors but absent or vestigial in rodents.

Comment 2: The statement that "Overall, the NHP retina exhibits larger photoreceptor inner segments (IS) and outer segments (OS) than the canine retina" requires clarification. It is unclear whether "larger" refers to photoreceptor length or diameter. In addition, the cited references do not directly support claims regarding photoreceptor cell size; rather, they discuss differences in overall retinal size and photoreceptor density, not photoreceptor cell size. Although NHP cones have been shown to be larger than those in rodents, the size of NHP rods appears comparable to that in rodents. Therefore, this interpretation should be supported by images from non-expanded samples.

Response: In the original text, “larger” referred to both the longitudinal length and transverse width/diameter of the photoreceptor IS/OS region in the sampled NHP retina compared with the canine retina. We agree that this point was not sufficiently clear. We also agree that the cited studies do not directly state that NHP photoreceptors are larger than those of other species, as direct comparison of photoreceptor cell size was not the primary focus of those reports. However, the histological images presented in these papers are consistent with species-dependent differences in photoreceptor IS/OS dimensions, particularly cone IS width in Grünert and Martin and IS/OS length in Booler et al.

© 2026. Published by The Company of Biologists under the terms of the Creative Commons Attribution License (https://creativecommons.org/licenses/by/4.0/).

To support this interpretation more directly, we have added representative images from non-expanded retinal sections stained with WGA and PNA (**Supplementary Fig. S3**). These images confirm that the larger IS/OS profiles observed in U-ExM samples are also evident in conventionally processed, non-expanded samples. In particular, they show that, compared with the canine retina, NHP photoreceptors have longer IS/OS profiles and greater transverse width/diameter at the IS level. We have revised the text to clarify that this statement refers to both IS/OS length and width/diameter, and we now present the non-expanded images as direct support for this comparison.

Line 123: In low-magnification U-ExM images, NHP photoreceptors showed larger IS/OS profiles than canine photoreceptors, including greater longitudinal length and transverse width/diameter in the sampled retinal region (Fig. 1B). This difference was also observed in non-expanded retinal sections, indicating that the apparent size difference was not generated by the expansion procedure (Supplementary Fig. S3). These observations are consistent with previous conventional histological studies showing larger primate photoreceptor IS/OS profiles compared with those of rodents and other mammals, including dogs (Booler et al., 2022; Grünert and Martin, 2020).

Line 757: Overall, NHP photoreceptors exhibit longer IS/OS profiles and greater transverse width/diameter at the IS level than canine photoreceptors. PSC axoneme is visualized by glutamylation immunolabeling (magenta).

Supplementary Fig. S3 legend: Non-expanded retinal sections showing photoreceptor IS/OS morphology in canine and NHP retinas. Representative single z-slice confocal images of non-expanded canine and NHP retinal sections stained with wheat germ agglutinin (WGA; gray) and peanut agglutinin (PNA; magenta). The field of view includes the outer limiting membrane (OLM), inner segment (IS), and outer segment (OS) regions, corresponding to the region shown in Fig. 1B. Lower panels show transverse views through the IS region. These images show that NHP photoreceptors have longer IS/OS profiles and greater transverse width at the IS level than canine photoreceptors. Scale bars, 10 µm.

Comment 3: The sentence "…the overall dimensions of the tubulin-based PSC scaffold appeared broadly similar between canine and NHP photoreceptors (Fig. 1C)" is difficult to interpret. First, the term "tubulin-based PSC scaffold" is not clearly defined. Second, Figure 1C does not show quantitative measurements supporting this conclusion. The authors should either define the structural feature being referenced and provide the corresponding quantification or revise the statement.

Response: We agree that the phrase “overall dimensions of the tubulin-based PSC scaffold” was not sufficiently precise and that Fig. 1C alone did not provide quantitative support for such a broad statement. Our intention was to indicate that, despite the larger IS/OS profiles observed in NHP photoreceptors, the basal microtubule-based ciliary structure was readily resolved in both canine and NHP photoreceptors and that the basal body/mother centriole axoneme width was comparable across species and between rods and cones. To avoid ambiguity, we have removed the phrase “tubulin- based PSC scaffold” and revised the text to restrict the statement of conservation to BB/mother centriole axoneme width, which is quantitatively supported by Supplementary Fig. S1. We then describe the differences in individual PSC subcompartments across species and between rods and cones based on the corresponding analyses.

Line 129: In contrast, BB axoneme width was comparable across species and between rods and cones, consistent with the use of this structure as an internal reference for expansion factor estimation (**Supplementary Fig. S1**). Closer examination of individual PSC subcompartments, however, revealed clear rod-cone variation and species-dependent differences.

Comment 4: Figure 2E. The cartoon indicates "Apical end of the CC" at position 0, whereas the legend states that "The distal end of the CC was used to define the 0 nm position." These descriptions

© 2026. Published by The Company of Biologists under the terms of the Creative Commons Attribution License (https://creativecommons.org/licenses/by/4.0/).

should be made consistent. In addition, the term "apical region of PSCs" is not properly defined. Logically, the apical region of the PSC should correspond to the outer segment tip.

Response: To avoid inconsistency and ambiguity, we have revised the label in Fig. 2E from "apical end of the CC" to "distal end of the CC," consistent with the figure legend. We have also replaced the term "apical region of PSCs" with "distal CC/proximal OS axoneme region". These revisions clarify that the 0 nm position refers specifically to the distal end of the connecting cilium, not to the apical tip of the entire photoreceptor sensory cilium/outer segment.

Comment 5: Figure 2F legend. The statement "The CC and axoneme were visualized by AcTub/SPATA7 immunolabeling" does not explain that SPATA7 labels the connecting cilium region. Please clarify which structure is labeled by each marker.

Response: We have revised the Figure 2F legend to clarify the structures labeled by each marker. Specifically, AcTub labels the microtubule-based axoneme, whereas SPATA7 labels the connecting cilium/Y-link-associated region. The revised legend now explicitly states this distinction.

Line 796: The ciliary axoneme was visualized by AcTub immunolabeling (magenta), and the connecting cilium/Y-link-associated region was visualized by SPATA7 immunolabeling (green). NHS-ester counterstaining (gray) was used as a pan-protein morphological reference to visualize surrounding photoreceptor structures.

Comment 6: Figure 3G presents cartoons summarizing bulge-region features in canine and NHP rods and cones. Please move this panel to Figure 2, as Figure 3 which focuses exclusively on human data.

Response: Although our intention was to use this panel as an integrated summary after presenting the human data, the schematic primarily summarized the canine and NHP PSC features established in Figs. 1 and 2. We have therefore moved this panel from Fig. 3 to Fig. 2, where it now serves as a schematic summary of the canine–NHP comparison of PSC architecture. We have also revised the corresponding figure legend and text to clarify that the panel summarizes the structural features supported by the canine and NHP analyses. The relationship of the human data to the NHP pattern is now described in the Fig. 3 Results section and Discussion rather than being incorporated into this schematic.

Comment 7: Figure 4D. The claim that in NHP rods the whirlin-positive structures are arranged as "pairs of adjacent pillar-like structures around the CC" is not supported by the presented images. Some of the whirlin structures in the NHP cells appear to be non-paired. The authors should either provide stronger visual support for this interpretation or modify the description.

Response: We agree with the reviewer. The original wording implied that all Whirlin-positive structures in NHP rods were consistently resolved as pairs of adjacent pillar-like structures, but this interpretation was not fully supported by a single axial optical section. We also agree that some Whirlin-positive structures can appear non-paired in individual optical sections.

In response, we have revised the manuscript to describe this pattern more cautiously. Specifically, we no longer state that all Whirlin-positive structures are arranged as paired elements. Instead, we now state that many individual Whirlin-positive elements in NHP rods can be resolved as adjacent paired pillar-like structures in at least some serial z-sections, while acknowledging that this paired appearance is not evident for every element in every optical section.

To provide stronger visual support, we have added Supplementary Fig. S6A, which shows serial axial-view z-section images through the CC/periciliary region of additional canine and NHP rod photoreceptors not shown in the main figures, with two representative cells shown for each group. These additional images support the revised interpretation that NHP rods show circumferential Whirlin-positive organization around the CC and that many individual Whirlin-positive elements can appear as adjacent paired pillar-like structures in some z-planes. We have also revised the main text to avoid implying a fixed number of Whirlin-positive units or universal paired organization across all elements.

© 2026. Published by The Company of Biologists under the terms of the Creative Commons Attribution License (https://creativecommons.org/licenses/by/4.0/).

Line 234: Three-dimensional reconstructions further revealed that Whirlin in canine rods covered only a restricted domain corresponding to the IS-facing side of the CC, whereas in NHP rods Whirlin- positive structures extended around the circumference of the CC (**Fig. 4B**). High-magnification axial imaging confirmed this difference in periciliary organization. NHS-ester counterstaining showed that the periciliary membrane in canine rods was confined to the IS-facing side of the CC, whereas in NHP rods it extended around the full circumference of the CC. Whirlin-positive elements were localized along these periciliary membrane domains. In NHP rods, many Whirlin-positive elements were resolved as adjacent paired pillar-like structures in at least some serial z-sections, although this paired appearance was not evident for every element in every optical section (**Fig. 4C, D; Supplementary Fig. S6A**).

Supplementary Fig. S6 legend: Serial z-section analysis of Whirlin-positive periciliary structures in canine and NHP photoreceptors. (A, B) Representative serial axial-view U-ExM images through the CC/periciliary region are shown for rods (A) and cones (B). Two representative photoreceptors are shown for each group: canine rods, NHP rods, canine cones, and NHP cones. In canine rods, Whirlin- positive structures were restricted to the IS-facing side of the CC, whereas in NHP rods, canine cones, and NHP cones, Whirlin-positive structures were distributed circumferentially around the CC. Many individual Whirlin-positive elements could be resolved as adjacent paired pillar-like structures in at least some z-sections, although this paired appearance was not evident for every element in every optical section. Scale bars, 500 nm, corrected for the expansion factor.

Comment 8: The authors claim that calyceal processes are rudimentary or absent in canine rods, in contrast to NHP rods. However, the PCDH15 and actin staining in Figure 5D appears similar in canine and NHP rods. This interpretation should therefore be reconsidered.

Response: We agree that the difference between canine and NHP rods is not readily apparent from the background rod photoreceptors shown in Fig. 5D. However, Fig. 5 primarily focuses on the organization of periciliary structures in cones, and the three-dimensional rendering in Fig. 5D was optimized to visualize the foreground cone. The rod photoreceptors visible in the background are located in different optical planes, making the spatial relationship between PCDH15-positive profilesand actin filaments difficult to assess in this panel. The conclusion regarding canine and NHP rods is instead primarily supported by the rod-focused analyses presented in Fig. 4, particularly Fig. 4F.

We also respectfully disagree that the presence of both PCDH15 and actin signals alone indicates equivalent calyceal-process organization. Calyceal processes are defined not simply by the presence of these molecular components, but by their characteristic spatial organization. In canine rods, actin filaments are detectable, but few are continuous with PCDH15-positive structures and presence of axially oriented PCDH15-positive structures are absent (Fig. 5D left). By contrast, NHP rods show prominent axially oriented PCDH15-positive profiles accompanied by actin filaments extending toward and along these structures (Fig. 5D right). This distinction is clear and consistent with Fig. 4F.

Thus, the relevant distinction is not the presence or absence of PCDH15 and actin signals themselves, but whether they form the spatially coordinated, axially oriented molecular organization characteristic of calyceal processes. Based on these findings, we retain our conclusion that calyceal processes are rudimentary or absent in canine rods, in contrast to the well-developed calyceal processes observed in NHP rods.

Comment 9: Figure 6A. The text states: "Actin filaments extending toward these PCDH15-positive structures were also evident, further supporting a NHP-like periciliary organization in human photoreceptors (Fig. 6A)." However, panel A does not include actin staining.

Response: We agree with the reviewer. This was an incorrect figure citation. The actin staining is shown in Fig. 6B, not Fig. 6A. We have corrected the text accordingly.

© 2026. Published by The Company of Biologists under the terms of the Creative Commons Attribution License (https://creativecommons.org/licenses/by/4.0/).

Comment 10: I have a significant concern about the quality of tissue preservation and/or processing shown in Figure 7A. Why are mitochondria not stained in two out of the three cells shown in panel "human replicate 1"? And why is cytochrome C brightly stained in one of the canine outer segments? These data do not meet the standard of publication quality.

Response: We respectfully note that the reviewer's interpretation of these panels appears to have been affected by the fact that individual photoreceptors are not perfectly aligned within retinal sections. The vertical position of the CC and OS base can vary slightly from cell to cell within the same field of view. This is not specific to U-ExM processing, but is also observed in conventional retinal tissue sections and EM images, as illustrated, for example, in Fig. 3 of Spencer et al. **(PMID: 31843915)** and in Fig. 29.1 of Lewis et al. **(PMID: 39930192)**.

With this point in mind, the two cytochrome c-negative profiles in the human replicate 1 panel do not represent IS regions lacking mitochondrial staining. Rather, these profiles correspond to OS regions, where mitochondria are not expected. Similarly, in the canine panel, the bright cytochrome c-positive structures noted by the reviewer are not located within the OS. Instead, they correspond to mitochondria in the IS region that appear at a similar vertical level to the CC because of the slight cell-to-cell offset in photoreceptor alignment. Thus, we do not think that these signals indicate poor tissue preservation or aberrant cytochrome c staining.

Nevertheless, we agree that the original figure presentation could cause confusion. To avoid misinterpretation, we have replaced these panels with clearer representative images.

Comment 11: Also, the authors state that no additional tubulin-based scaffold was detected on the side of the IS opposite the ciliary axoneme, in contrast to a prior EM study. However, tubulin staining is clearly present within the accessory inner segment in Human replicate #2. The authors should address and reconcile this inconsistency.

Response: We agree that our original statement was misleading. In particular, it was not appropriate to state that "no additional tubulin-based scaffold was detected on the side of the IS opposite the ciliary axoneme," because tubulin signal is indeed present within the aIS-like structures in human replicate 2.

We have therefore reconsidered our interpretation of the tubulin labeling in this region. Lewis et al. (2025) described the aIS as an IS-derived structure containing non-ciliary microtubules and therefore did not claim that the aIS-associated tubulin scaffold is identical to the ciliary axoneme. At the same time, their study strongly emphasized the aIS as a unique structure reinforced by a dense and extensive microtubule-based cytoskeleton. Moreover, the β-tubulin immunofluorescence image shown in Fig. 2b of Lewis et al. could be interpreted as showing a prominent tubulin-positive structure on the side of the IS opposite the ciliary axoneme, giving the visual impression of an aIS-associated scaffold that is highly developed and comparable to the signal patern of ciliary axoneme. In our U- ExM images, however, the tubulin-positive signal within the aIS-like region appeared weaker and more variable than that of the ciliary axoneme, and identical with the broader IS tubulin cytoskeleton. Thus, our revised interpretation is not that tubulin is absent from the aIS-like structure, but that the tubulin-positive elements observed in the aIS-like structure appear to represent an extension of the broader IS microtubule network rather than a discrete scaffold unique to the aIS-like region.

To address this point more carefully, we performed additional imaging of human retinal samples using the same anti-β-tubulin antibody used in the original experiment and an additional anti-α-tubulin antibody. These new data are now presented in Supplementary Fig. S8. Under our U-ExM conditions, the anti-α-tubulin antibody provided clearer labeling of both the ciliary axoneme and the IS microtubule cytoskeleton than the anti-β-tubulin antibody. In human replicate 1, we did not observe prominent aIS- like structures or associated tubulin-positive extensions in rods or cones. In human replicate 2, aIS- like structures were observed in several rods and cones, and both β-tubulin and α-tubulin signals were detected within these regions. However, these signals were variable and uneven and appeared identical with the broader IS tubulin network rather than forming a discrete scaffold unique to the aIS- like region.

© 2026. Published by The Company of Biologists under the terms of the Creative Commons Attribution License (https://creativecommons.org/licenses/by/4.0/).

Based on these additional data and our re-evaluation of the original images, we have revised the manuscript to state that aIS-like structures in human replicate 2 contain tubulin-positive cytoskeletal elements. We now interpret these tubulin-positive elements as being more consistent with an extension of the IS microtubule cytoskeleton than with a separate, uniquely organized tubulin scaffold within the aIS-like structure.

Line 324: Tubulin immunolabeling further showed that aIS-like structures in human replicate 2 contained α-tubulin- and β-tubulin-positive signals, but these signals were variable and uneven, resembling the broader IS microtubule network rather than forming a discrete scaffold unique to the aIS-like region (**Fig. 7A; Supplementary Fig. S8**).

Line 333: As in rods, these structures were supported by morphological features visualized by NHS-ester staining, the presence of cytochrome c-positive mitochondria within the OS-associated extension, tubulin-positive signals resembling the broader IS microtubule network, and an elongated PCDH15-positive IS/OS junction (**Fig. 7B; Supplementary Fig. S8**).

Supplementary Fig. S8 legend: Tubulin organization in human accessory inner segment-like structures. **(A, B)** Representative single optical section images of human rod (A) and cone (B) photoreceptors showing the distribution of IS mitochondria visualized by cytochrome c immunolabeling (cyan) and the microtubule cytoskeleton visualized by β-tubulin (upper panels) or α- tubulin (lower panels) immunolabeling (magenta). In human replicate 1, aIS-like structures and associated tubulin-positive extensions were not observed in either rods or cones. In human replicate 2, several rods and cones showed mitochondria-containing aIS-like structures extending alongside the OS. Both β-tubulin- and α-tubulin-positive signals were detected within these aIS-like regions, but the signals appeared variable and uneven, resembling the broader IS microtubule network rather than a scaffold unique to the aIS-like structure. One representative cell is shown for each staining condition and rods and cones in human replicate 1, and two representative cells are shown for each staining condition and rods and cones in human replicate 2. All images are shown as single optical sections. Scale bars, 1 µm, corrected for the expansion factor. All microscopy images in this supplementary figure are U-ExM images.

Reviewer #2

Major comment 1: Needed are discussions of the possible functional significance of longer daughter centrioles in NHP cones, the shortening of CCs in NHPs compared to canines, and the unique whirlin distribution in NHP rods.

Response: We agree with the reviewer that the possible functional significance of these structural differences should be discussed more explicitly. Rather than adding a single speculative paragraph, we have incorporated focused discussion at the relevant points in the Discussion, as described below. We have been careful to present these points as possible implications rather than definitive mechanistic conclusions, because the direct functional consequences of these architectural differences remain to be tested experimentally.

Line 403: In primate photoreceptors, the presence of larger and more elongated IS/OS profiles may favor a shorter CC as a more compact supporting segment between the IS and the enlarged OS. In parallel, the expanded bulge region in NHP and human photoreceptors may reflect the need for a more developed proximal OS axonemal region at the site where the larger OS is initiated and organized. Thus, the shorter CC and larger bulge region observed in primates may represent coordinated structural adaptations that help stabilize and organize larger photoreceptor OS compartments. The functional significance of DC elongation is more difficult to infer, because the role of the DC in mature photoreceptor cilia remains poorly understood. We therefore avoid assigning a specific mechanistic role to this feature. Nevertheless, the pronounced elongation of DCs in primate cones may represent another aspect of primate cone specialization, potentially related to the enlarged cone IS compartment and the organization of the basal ciliary apparatus.

© 2026. Published by The Company of Biologists under the terms of the Creative Commons Attribution License (https://creativecommons.org/licenses/by/4.0/).

Line 432: The circumferential Whirlin-positive organization in primate rods may also provide a structural context for the phenotypic gap between human USH2-associated retinopathy and mouse USH2 models. In humans, USH2-associated retinal disease manifests as progressive retinitis pigmentosa, with progressive visual field constriction and eventual severe visual impairment in many patients (Iannaccone et al., 2004; Pierrache et al., 2016). By contrast, mouse models affecting USH2 proteins generally show mild or very late-onset retinal degeneration compared with the human disease course (Liu et al., 2007; Yang et al., 2010; Lewis et al., 2025). Our data suggest that this phenotypic discrepancy may partly reflect species-dependent differences in rod periciliary architecture. In primate rods, the Whirlin-positive elements surround the CC in a circumferential, cone- like manner, suggesting that the USH2-associated periciliary membrane may contribute more broadly to structural support of this region. By contrast, in rods with a more restricted periciliary domain, such as rodent and canine rods, the CC may be less dependent on USH2-associated periciliary support. This could make the structural consequences of USH2 disruption less severe or more slowly progressive in mouse models than in primate photoreceptors. This idea may also be relevant to canine inherited retinal disease (IRD) genetics. Despite the large number of naturally-occurring canine IRD rods raises the possibility that, as in rodents, canine rods may be relatively less vulnerable to disruption of USH2-associated periciliary support than primate rods. Although this model remains speculative and requires direct experimental testing, it provides a testable structural hypothesis linking species-specific periciliary architecture to the incomplete recapitulation of human USH2 retinopathy in non-primate animal models.

Major comment 2: Overall the abstract is esoteric and requires knowledge of cilia and photoreceptor ultrastructure. Incorrect or confusing terminology is used that should be clarified. The abstract should also describe the key structural and functional importance of the connecting cilium and periciliary region and describe their relevance to retinal disease.

Response: We agree that the original Abstract assumed substantial prior knowledge of photoreceptor ciliary anatomy and included terminology that could be unclear to readers outside this field. We have therefore substantially revised the Abstract to improve its accessibility and anatomical precision. We have also replaced ambiguous or highly specialized terminology with clearer descriptions, as detailed in our responses to Detailed comments 1–4 below.

The present study focuses on comparative nanoscale architecture and molecular organization and does not directly examine functional consequences or retinal disease mechanisms. Given the 180-word limit, we have therefore prioritized the anatomical context and findings directly supported by the present data, rather than adding broader statements regarding function or disease relevance that were not experimentally tested in this study. Possible functional and disease-related implications are discussed where appropriate in the main text.

Major comment 3: Fig. 1E and Fig. 2A/B depict results from similar analyses, yet the measurements and some of the comparisons draw different conclusions. For example, canine cone CC length is (presumably) not significantly different from NHP rod CC length in Fig. 2C. These differences in the data should be explored and discussed. Ideally, the datasets should be presented together to assist interpretation. Furthermore, the same canine measurements were performed in Takahashi et al. (2025). Yet, there seems to be some variability in the measurements from Fig. 3H, 5K, and 6S of the 2025 paper compared to Fig. 1E of this manuscript. Please indicate that the same differences were replicated/confirmed between studies.

Response: The biological conclusions drawn from these analyses are the same. Both datasets support two main points: first, rods have longer CC-associated structures than cones within both canine and NHP retinas; and second, canine photoreceptors have longer CC-associated structures than NHP photoreceptors within both rods and cones. We did not intend to emphasize cross-category comparisons that combine both species and photoreceptor types, such as canine cones versus NHP rods. The phrase “with canine rods showing the longest and NHP cones the shortest values” was intended only as a descriptive summary of the overall range, but we agree that it could be interpreted as emphasizing such cross-category comparisons. To avoid this confusion, we have removed this phrase.

Regarding the comparison with Takahashi et al. (2025), we agree that the canine measurements in the present study differ modestly from some values reported in our previous paper. This difference

© 2026. Published by The Company of Biologists under the terms of the Creative Commons Attribution License (https://creativecommons.org/licenses/by/4.0/).

is most likely explained by retinal sampling region. In the previous study, canine measurements were obtained from multiple retinal regions without strict regional standardization and were derived mainly from mid-peripheral to peripheral retinal areas. In the present study, we specifically evaluated regional effects on canine CC length and found that CCs were modestly but significantly longer in the superior central retina than in the peripheral retina. The peripheral-region values in the present dataset were broadly consistent with the values reported in Takahashi et al. (2025), indicating that the difference between studies primarily reflects retinal topography and sampling strategy rather than lack of reproducibility. We have clarified this point in the 2nd paragraph of the Results section.

Major comment 4: Line 178: The conclusion that human rod and cone CCs were shorter than canine and "more closely resemble" NHP CCs is vague. Why aren't all the human vs. NHP vs. canine measurement data directly compared? These are the truly novel comparisons in this work. For this reason, the data in Supplementary Figure 4A should be presented in the main figure.

Response: We agree that the human data are important and that the wording “more closely resembled NHP photoreceptors” was somewhat vague. We have therefore revised this statement to clarify that this was intended as a descriptive comparison, not as a strictly matched statistical comparison across all three species.

However, we respectfully disagree that all human, canine, and NHP measurements should be directly compared in the same way as the canine–NHP dataset. The canine and NHP samples were obtained from young adult animals and were processed under standardized fixation and cryopreservation conditions. By contrast, the human donor samples were derived from elderly donors and differed substantially in postmortem interval, fixation duration, and overall tissue condition. In addition, the two human biological replicates showed noticeable differences in ciliary axoneme morphology, and similar donor-to-donor variability was also evident in the subsequent analysis of periciliary structures. For these reasons, we intentionally treated the canine–NHP comparison as the primary controlled cross- species comparison and used the human data to assess whether the structural features identified in canine and NHP photoreceptors were broadly reflected in human photoreceptors. To make this rationale clearer, we have added a sentence to the Results section explaining that the human data were interpreted descriptively and with caution because of these sample differences.

Regarding Supplementary Fig. S4A, we agree that this dataset is informative. However, this analysis addresses a specific, limited question: whether CC and DC length differ between S-cones and L/M-human-canine-NHP comparisons are limited by the sample differences described above, we have retained this dataset as a supplementary figure. We believe that this presentation preserves the main structure of the manuscript while still making the cone-subtype analysis available to readers.

Line 180: For this reason, the human dataset was analyzed separately from the controlled canine-NHP comparison and interpreted descriptively, rather than as part of a strictly matched cross-species comparison.

Line 191: When the combined human data were compared descriptively with the canine and NHP datasets, human rod and cone CC lengths were shorter than the corresponding canine values, showing a pattern consistent with the shorter CC lengths observed in NHP compared with canine photoreceptors for both rods and cones (**Fig. 3C**).

Major comment 5: Line 220: There are six whirlin puncta in the example in Fig. 4B, but are there always six? The rod in Fig. 4C may have more than six. This localization should be quantified. Please provide the full Fig. 4A-C dataset of images, from which this important conclusion is derived.
(related) Fig. 5B,C: The pillars, again, should be quantified and compared. It would be specifically interesting to compare them between canine cones and NHP rods.
·(related) Line 257: The conclusion of five pairs vs. six pairs is potentially of notable, functional significance but cannot be broadly made without more examples or, preferably, quantification with statistics.

Response: We agree that the original wording, particularly the statements referring to six

© 2026. Published by The Company of Biologists under the terms of the Creative Commons Attribution License (https://creativecommons.org/licenses/by/4.0/).

Whirlin- positive elements in NHP rods and five versus six paired elements in cones, implied a level of numerical precision that was not sufficiently documented in the original figure presentation.

In response to this comment, we attempted to quantify the number of Whirlin-positive puncta/pillar- like structures around individual PSCs. However, we found that reliable counting was not feasible with the current imaging dataset. The apparent number of Whirlin-positive puncta varied depending on z- position, local signal quality, imaging angle, and the extent to which adjacent puncta were resolved as separate structures or partially merged within a given optical section. In particular, paired or adjacent paired pillar-like configurations were often visible in at least one z-plane, but not all Whirlin- positive elements could be consistently classified as paired structures across serial optical sections. We therefore concluded that presenting formal numerical counts or statistical comparisons would risk overinterpreting the data.

To address the reviewer's concern while avoiding unsupported quantification, we have added Supplementary Fig. S6, which shows serial z-slice images from additional photoreceptors not shown in the main figures. This includes two additional examples each of canine rods, NHP rods, canine cones, and NHP cones. These additional images provide broader visual support for the revised conclusion that Whirlin organization differs primarily in its restricted versus circumferential distribution

We have also revised the manuscript to remove specific numerical claims regarding six versus five Whirlin-positive units. The revised text now focuses on the more robust structural conclusion: canine rods show a restricted, non-circumferential Whirlin-positive domain along the IS-facing side of the CC, whereas NHP rods, canine cones, and NHP cones show a circumferential Whirlin-positive organization around the CC. We also state more cautiously that adjacent paired pillar-like Whirlin- positive structures were frequently observed in photoreceptors other than canine rods, but that not every punctum could be unambiguously resolved as a paired structure in every z-section.

We agree that comparison between canine cones and NHP rods could be biologically interesting. However, because the exact number of Whirlin-positive units could not be robustly quantified, we have avoided drawing conclusions based on numerical comparisons between these groups. Instead, we now focus on the reproducible qualitative feature that both canine cones and NHP rods show circumferential Whirlin-positive organization, in contrast to the restricted distribution observed in canine rods.

Line 234: Three-dimensional reconstructions further revealed that Whirlin in canine rods covered only a restricted domain corresponding to the IS-facing side of the CC, whereas in NHP rods Whirlin- positive structures extended around the circumference of the CC (**Fig. 4B**). High-magnification axial imaging confirmed this difference in periciliary organization. NHS-ester counterstaining showed that the periciliary membrane in canine rods was confined to the IS-facing side of the CC, whereas in NHP rods it extended around the full circumference of the CC. Whirlin-positive elements were localized along these periciliary membrane domains. In NHP rods, many Whirlin-positive elements were resolved as adjacent paired pillar-like structures in at least some serial z-sections, although this paired appearance was not evident for every element in every optical section (**Fig. 4C, D; Supplementary Fig. S6A**).

Line 269: High-magnification axial imaging and serial z-section analysis showed that Whirlin-positive structures were distributed circumferentially around the CC in both canine and NHP cones. Within this circumferential array, many individual Whirlin-positive puncta could be resolved into adjacent paired pillar-like elements, although this paired appearance was not evident for every element in every optical section **(Fig. 5B, C; Supplementary Fig. S6B**).

Supplementary Fig. S6 legend: Serial z-section analysis of Whirlin-positive periciliary structures in canine and NHP photoreceptors. **(A, B)** Representative serial axial-view U-ExM images through the CC/periciliary region are shown for rods (A) and cones (B). Two representative photoreceptors are shown for each group: canine rods, NHP rods, canine cones, and NHP cones. In canine rods, Whirlin- positive structures were restricted to the IS-facing side of the CC, whereas in NHP rods, canine cones, and NHP cones, Whirlin-positive structures were distributed circumferentially

© 2026. Published by The Company of Biologists under the terms of the Creative Commons Attribution License (https://creativecommons.org/licenses/by/4.0/).

around the CC. Many individual Whirlin-positive elements could be resolved as adjacent paired pillar-like structures in at least some z-sections, although this paired appearance was not evident for every element in every

Major comment 6: Fig. 7B: It is unclear from these cone examples if the human cones have aIS-like extensions. The top human replicate 2 cone shows mitochondrial staining in this region above the BB, but it is not evidently adjacent to the OS, and so this could represent an extension of the IS proper. In the second human replicate 2 cone, the indicated region looks like the periciliary regions highlighted with Fig. 6. Thus, the aIS distinction requires mitochondrial labeling and clear apposition to the OS from the same cell (as shown for rods in Fig. 7A). Can such an example be provided for cones?

Response: We have replaced the human replicate 2 cone example in Fig. 7B with a clearer image. In the revised panel, cytochrome c-positive mitochondria are present within an IS-derived extension that is directly apposed to the OS in the same cone photoreceptor.

Major comment 7: Line 337/Fig. 8B: There are no apparent ciliary rootlet differences among the different cone examples presented here. Each of the examples could be considered branched and curvilinear. Please provide more examples or measurements. Otherwise, the strong conclusions about rootlet differences should be removed from the text.

Response: We agree that the original wording did not sufficiently clarify the specific rootlet feature that differs between canine and primate cones. Our intended conclusion was not based simply on whether the rootlet appeared curved or branched, because some degree of curvature or branching can indeed be appreciated in all cone examples. Rather, the key difference concerns the terminal organization of the cone ciliary rootlet.

In canine cones, consistent with our previous canine U-ExM study (Takahashi et al., 2025), the rootlet typically forms a curvilinear arc in which the distal/rootlet terminal portions remain connected, creating a continuous curved configuration. By contrast, in NHP and human cones, the rootlet termini appear separated into two or three thin fibers that extend toward the basal IS region rather than forming a continuous arc-like terminal structure. We have revised the Results text to define this distinction more explicitly.

To provide stronger visual support, we have added Supplementary Fig. S9 showing additional cone examples not used in Fig. 8B. This includes two additional cones each from canine, NHP, human replicate 1, and human replicate 2 retinas. These additional examples support the revised interpretation.

Line 351: whereas canine cones displayed a curvilinear rootlet architecture in which the terminal portions remained connected, forming a continuous arc-like configuration within the IS

Line 361: Unlike canine cones, in which the rootlet terminal portions remained connected in a continuous arc-like configuration, NHP and human cones appeared to show separated rootlet termini extending as two or three thin fibers. These fibers did not appear to form a continuous arc-like terminal structure and instead appeared to extend into the intermediate region of the IS (**Fig. 8B; Supplementary Fig. S9**).

Supplementary Fig. S9 legend: Additional examples of cone ciliary rootlet organization across species. Representative U-ExM images of cone photoreceptors immunolabeled for rootletin, showing additional examples not included in Fig. 8B. Two cone photoreceptors are shown for each group: canine, NHP, human replicate 1, and human replicate 2. In canine cones, the terminal portions of the ciliary rootlet form a continuous arc-like configuration. In NHP and human cones, the rootlet termini are separated into thin fibers extending toward the basal IS region. Scale bars, 1 µm, corrected for the expansion factor.

Detailed comment 1: Line 30, first sentence: This is a confusing start: Photoreceptor sensory cilia (PSC) contain the OS, along with the BB, CC, and axonemes within, and so it should not be written that the PSC supports outer segment formation; rather, the PSC includes the structures that support the proper formation (i.e., the axoneme and BB centrioles) and organization of outer

© 2026. Published by The Company of Biologists under the terms of the Creative Commons Attribution License (https://creativecommons.org/licenses/by/4.0/).

segment discs. The "PSC" terminology, while consistent with the author group's past literature, should be reconsidered in some cases or at least explained for the sake of new readers not familiar with the past papers, such as experts in other types of cilia.

Response: Because Detailed comments 1–4 all concern the Abstract, we provide the revised Abstract and a consolidated response to the corresponding textual revisions after Detailed comment

4. The concern raised here regarding the structural relationship between the photoreceptor cilium and the outer segment is addressed in that consolidated response. Here, we respond specifically to the terminology "PSC."

We agree that this terminology should be clearly explained for readers who may be unfamiliar with the photoreceptor cilium literature. However, we respectfully note that "PSC" are not terminology unique to our group, but have been used in multiple previous publications of photoreceptor ciliary biology (**PMID: 17494944, 20238021, 26068394**). We have therefore retained the term in the main text, where it is explicitly defined, but have avoided using the abbreviation "PSC" in the revised Abstract to improve accessibility for a broader readership.

Detailed comment 2: Line 31, still the first sentence: The term "subtype-dependent organization" is also confusing without any additional context. This terminology is used again on line 34, and it is used throughout the group's papers. I would suggest orienting readers to indicate the meaning of subtype (as in Takashi et al. 2025), which, in the context of this manuscript, refers to the major photoreceptor cell type, more specifically, rods vs. cones. On, for example, lines 157 and 163 (but also elsewhere in the manuscript), the authors use the term "rod-cone differences," which is preferable, clearer, and better aligned with the literature. Such terminology vagaries are potentially exacerbated by suggestions from a writing tool that is incompletely trained on the appropriate literature. In general, more cautious evaluation of the terms and wording provided from such tools may be needed.

Detailed comment 3: Line 36: The "bulge region" is also confusing without any context or prior knowledge (see below for more about this term).

Detailed comment 4: Line 44: "Principles" is another confusing term when describing cell structures. It could be replaced with something more clear, like "structural features."

Response: In response to Detailed comments 1–4, we have comprehensively rewritten the Abstract. The revised version clarifies the anatomical relationship among the basal body, connecting cilium, ciliary axoneme, and outer segment; introduces the location of associated periciliary structures; retains the full term "photoreceptor sensory cilia" while avoiding the abbreviation "PSC"; replaces "subtype-dependent" with explicit rod-cone terminology; removes the specialized term "bulge region"; and replaces "principles" with more precise structural and molecular terminology. We have also standardized the expression "periciliary structures" throughout the Abstract to avoid ambiguity regarding the number or organization of periciliary complexes. The revised Abstract reads as follows:

Line 29: Photoreceptor sensory cilia comprise a microtubule-based structural core and the phototransductive outer segment. The ciliary axoneme extends from the basal body through the connecting cilium into the outer segment, thereby linking the inner and outer segments. Around this central axis, specialized periciliary structures are distributed across the apical inner segment and basal outer segment. Yet how these ciliary and periciliary structures differ between rods and cones and across mammals remains incompletely defined. Using ultrastructure expansion microscopy, we compared their nanoscale molecular organization across canine, non-human primate (NHP), and human retinas. Key rod-cone differences in ciliary organization were broadly conserved among all three species, whereas the scale and morphology of individual ciliary subcompartments differed between species. Periciliary structures showed even greater species-specific diversification, particularly in rods. Human photoreceptors shared several structural features with NHP photoreceptors but also displayed inter-individual variability in accessory inner segment-like structure. These findings refine current understanding of photoreceptor sensory cilia and associated periciliary structures in large mammals, revealing that conserved rod-cone

© 2026. Published by The Company of Biologists under the terms of the Creative Commons Attribution License (https://creativecommons.org/licenses/by/4.0/).

differences coexist with pronounced species-specific structural and molecular specializations.

Detailed comment 5: Lines 50-53: The "tubulin"/microtubule core should be distinguished as the axoneme in this section since this is the prevalent terminology. The CC has an axoneme that continues into the OS, and so the axoneme is part of both compartments.

Detailed comment 6: Line 53: The "bulge region," defined as such in Mercey et al. (2022), should be characterized with a description. While "bulge region" may serve as a technical term for the structure, it should be redefined in this manuscript for the unfamiliar. Others may understand it as the portion of the axoneme in the nascent disk region/OS base/proximal OS.

Response: We agree with the reviewer that the ciliary axoneme should be explicitly distinguished from the broader microtubule-based structural core. We have retained the broader microtubule-based structural core because the DC, BB, and ciliary axoneme are analyzed together throughout this study, but have revised the text to clarify their anatomical relationships. Specifically, we now state that the ciliary axoneme extends from the BB through the CC and continues into the OS. We have also defined the "bulge region" for readers who may be unfamiliar with this terminology. It is now described as the widened portion of the ciliary axoneme located immediately distal to the CC at the OS base. In addition, we have clarified the functional relationship between the microtubule-based structural core and the OS by noting that the CC provides the route through which the continuous supply of membrane and proteins required for OS renewal is delivered from the IS. Together, these revisions more clearly distinguish the centriolar structures from the axoneme, define the bulge region anatomically, and explain how these components form an integrated framework supporting OS formation and maintenance in rods and cones.

Line 51: In both rod and cone photoreceptors, the PSC comprises a microtubule-based structural core together with the outer segment (OS), a highly specialized membrane organelle dedicated to phototransduction. The microtubule-based structural core includes the daughter centriole (DC), basal body (BB), and ciliary axoneme, which extends from the distal end of the BB through the connecting cilium (CC) and continues into the OS. Immediately distal to the CC, the ciliary axoneme widens at the OS base, adjacent to the site of nascent disc or lamella formation in the proximal OS, forming a domain termed "the bulge region" before continuing as the distal axoneme (Faber et al., 2023; Mercey et al., 2022). The OS contains stacked membrane lamellae or discs that undergo continual renewal, requiring a substantial and continuous supply of membrane and proteins from the inner segment (IS) through the CC (Barnes et al., 2021). Together, the DC, BB, and ciliary axoneme form an integrated ciliary framework that links the IS to the membrane-rich OS and supports OS formation and maintenance.

Detailed comment 7: Line 57: The writing here suggests that the periciliary region surrounds the apical inner segment. I think the meaning is that that periciliary region is located at the apical IS or is part of it.

Response: We removed "the base of the OS and" from this sentence. However, as written in the following sentences, please note that we consider the IS/OS junction and the calyceal processes surrounding the base of the OS to be an extension of the periciliary membrane region in the apical IS described here, and we regard these collectively as the photoreceptor periciliary structures.

Detailed comment 8: Line 58: The ciliary pocket resembles such a structure in primary cilia, but the periciliary region does not (it is unique to photoreceptors). As a note: the periciliary membrane complex (PMC) abbreviation, which is familiar to some, could also create some unintended confusion with the "PSC" abbreviation.

Response: We respectfully note that the term "periciliary membrane" is also used in the context of "primary cilia" to describe the membrane region at the base of the cilium that connects the ciliary membrane with the plasma membrane and can invaginate to form the ciliary pocket **(PMID: 30733609, 37072495)**. Thus, although photoreceptor periciliary structures are specialized, the idea of a periciliary membrane adjacent to the ciliary pocket is not unique to photoreceptors.

To avoid overstatement and potential confusion, we have revised the text to clarify that the ciliary pocket/periciliary membrane region in photoreceptors shares conceptual similarities with

© 2026. Published by The Company of Biologists under the terms of the Creative Commons Attribution License (https://creativecommons.org/licenses/by/4.0/).

membrane subdomains at the base of primary cilia, whereas calyceal processes and the elaborated photoreceptor periciliary structure represent photoreceptor-specialized structures.

Line 63: In the periciliary region surrounding the apical IS, the PSC is accompanied by plasma membrane specializations, including a ciliary pocket and adjacent periciliary membrane domain (Sánchez-Bellver et al., 2021; Wensel et al., 2021). Although the ciliary pocket/periciliary membrane region shares conceptual similarities with membrane subdomains at the base of primary cilia (Anvarian et al., 2019; Mill et al., 2023), the elaborated photoreceptor periciliary structures, including the IS/OS junction and calyceal processes, represents a photoreceptor-specialized architecture.

Detailed comment 9: Line 62: The USH proteins and Usher syndrome (or any disease relevance of the work) are not properly introduced. Also, please indicate which species and cell types have the described USH protein distributions.

Response: We have revised the Introduction to state that Usher syndrome (USH)-associated proteins are disease-associated proteins that also serve as useful molecular markers of photoreceptor periciliary organization.

Because the main focus of this manuscript is not Usher syndrome pathogenesis, and because the reported localization of individual USH proteins varies depending on species, rod/cone, and periciliary subdomain, we have avoided adding an extensive disease-focused or species-by-species review in the Introduction. Instead, we have provided a concise introduction to the marker logic used in this study and address the possible disease relevance of species-dependent Whirlin-positive periciliary organization in the Discussion, as described in our response to Reviewer #2 Major Comment 1.

Line 68: These periciliary domains are further distinguished by the compartmentalized distribution of Usher syndrome (USH)-associated proteins, which are disease-associated proteins but also serve as useful molecular markers of photoreceptor periciliary organization. In many vertebrate photoreceptors, USH type 2 (USH2)-associated proteins localize to the periciliary membrane region, whereas USH type 1 (USH1)-associated proteins are enriched at the IS/OS junction and/or calyceal process- associated membrane contacts (El-Amraoui and Petit, 2014; Patel et al., 2026; Sahly et al., 2012; Takahashi et al., 2025).

Detailed comment 10: Line 66: Here it is indicated that there are multiple periciliary complexes.

Response: In responses to earlier reviewer's comments, the sentence containing this potentially confusing wording has been deleted.

Detailed comment 11: Line 67: This final sentence of the paragraph is misleading. In fact, dissecting the rod-cone and species differences in the cilia is a hot topic in the literature, and there is already published detailed knowledge, including among the cited references (Takahashi et al. 2025; Sahly et al. 2012; Verschueren et al. 2022; Lewis et al. 2025; etc.).

Response: We revised this sentence as follows: Accumulating evidence from studies of these specialized photoreceptor structures has begun to reveal rod-cone variation and species-dependent differences at the ultrastructural and molecular levels, raising the question of how these architectural features are conserved or diversified across mammals.

Detailed comment 12: Line 91: The title of the section is confusing. How is the PSC architecture conserved if there are species-specific differences?

Response: We have revised the section title as follows: "PSC architecture shows shared rod-cone patterns and species-dependent features in canine and NHP photoreceptors."

Detailed comment 13: Line 94: More terminology uncertainty: the standard meaning of "basal body"

© 2026. Published by The Company of Biologists under the terms of the Creative Commons Attribution License (https://creativecommons.org/licenses/by/4.0/).

in the field is mixed. In Gilliam et al. (2012), both centrioles were termed "BBs." Whereas in Wensel et al. (2021) and Mercey et al. (2022), they are distinguished as the mother centriole "MC" and "DC." Please provide a description of the BB region as the region comprising the MC, the DC, and the surrounding area that contains pericentriolar satellites, and then specify that the MC is interchangeably labeled as the "BB" in this manuscript.

Response: We agree that terminology for the basal region of photoreceptor sensory cilia is not completely uniform across the photoreceptor literature. In particular, some photoreceptor studies have used “basal bodies (BBs)” to refer broadly to the centriolar pair and “mother centriole (MC)” for the ciliary basal body. However, in the broader context of ciliary biology, the basal body (BB) is generally understood as the MC-derived ciliary basal structure that nucleates the ciliary axoneme, and is distinguished from the adjacent daughter centriole (DC). This convention is described in many review papers of ciliary biology **(PMID: 21536747, 27350441, 30733609, 37072495, 33748110, 41140281, 40729936)**. We have therefore retained the BB/DC terminology used in the present manuscript, which is also consistent with the terminology adopted in our previous publication after nomenclature was carefully considered during peer review (Takahashi et al., 2025).

We also note that the anatomical definitions of CC, BB, and DC in the present manuscript are explicitly illustrated in Fig. 1A. In this schematic, the CC, BB, and DC are shown as distinct components of the PSC, making clear which structures these terms refer to in this study. Therefore, we do not think it is necessary to state that the structure termed MC in some other studies is referred to as BB in the present manuscript. Nevertheless, to further reduce ambiguity for readers from different fields, we have added a brief clarification in the revised Fig.1 legend that, “In this manuscript, basal body (BB) refers to the mother centriole-derived ciliary basal part, whereas daughter centriole (DC) refers to the adjacent centriole.”

Detailed comment 14: Line 101: The expansion factor calculations, although based on a published method, seem to be error-prone. The range of measured values in Supplementary Figure 1C is 0.6 - 1 micron. This is a potential cell-to-cell EF variation of 2.6 - 4.3. Could the authors comment on the feasibility of optimization in which the EF is calculated individually for each cell by, for example, measuring the MC/BB width from each cilium for normalization to the standard? This seems to be important for accuracy, considering the aforementioned cell-to-cell variation and possibility of ongoing expansion during the imaging period. Related, Line 512: Please provide additional details of the EF calculation. Were FWHM intensity values from line scans used to calculate the MC/BB widths?

Response: We agree that expansion factor estimation is a critical consideration for quantitative U-ExM analysis. However, we respectfully note that the measured BB/MC width in U-ExM samples reflects a combination of factors, including potential local variation in expansion, biological variation in the native BB/MC axoneme width, and measurement variability. Therefore, the range of BB/MC widths observed in expanded samples should not be interpreted solely as cell-to-cell variation in EF.

Published ultrastructural measurements generally report mean ± SD rather than raw minimum-maximum values, but the reported SD itself indicates that native BB/MC width measurements contain biological and/or measurement variability (**PMID: 1201585, 31691972**). Depending on sample size, the expected range of individual measurements can be several-fold larger than the SD. Although we could not identify a suitable reference reporting raw minimum–maximum values specifically for BB/MC width, Potter et al. (2021) provide an illustrative example in which EM-based measurements of CC axoneme width in WT mouse retina show an approximately 80 nm minimum-to-maximum range in the plotted values (**Fig. 9J; PMID: 34520396**). While CC axoneme width is not identical to BB/MC width, this example illustrates that a measurement range of this order can occur in ultrastructural datasets independently of expansion. In our dataset, the expanded BB/MC width range highlighted by the reviewer is approximately 400 nm. Dividing this value by the average EF of 3.64 gives an estimated pre-expansion-equivalent range of approximately 110 nm. Thus, this degree of variation in expanded BB/MC width cannot be attributed solely to EF variability.

Nevertheless, we fully agree with the reviewer that the accuracy and reliability of EF estimation

© 2026. Published by The Company of Biologists under the terms of the Creative Commons Attribution License (https://creativecommons.org/licenses/by/4.0/).

are important for dimension measurements in expansion microscopy. At present, however, accurate cell-by-cell EF estimation remains technically challenging. Calculating an EF for each individual cilium would require assuming that every BB/MC has an identical native width and that all variation in measured expanded BB/MC width reflects local expansion variability. This assumption is unlikely to be valid and could instead propagate measurement noise and/or true biological variation into all other measurements from the same cell. As a fact, many prior photoreceptor U-ExM studies have used a dataset-level or experiment-level EF correction strategy similar to ours, rather than applying individual cell-specific EF values (**Faber et al., 2023; Mercey et al., 2024; Mercey et al., 2022; Moye et al., 2025**).

Importantly, we do not treat EF-corrected U-ExM measurements as being equivalent to direct measurements from non-expanded samples. Instead, we use these values primarily for relative comparisons among samples processed using standardized U-ExM preparation, imaging conditions, EF estimation strategy, and measurement criteria. Under these controlled conditions, we believe that the corrected measurements provide useful and reasonably reliable support for relative comparisons across species and between rods and cones, even though absolute values should be interpreted with appropriate caution. In the manuscript, we therefore use these measurements mainly to compare groups, and we do not overinterpret the absolute validity of each corrected value. All measurement values are provided in the supplementary data for transparency.

In response to the reviewer's comment, we have added a statement to the Discussion emphasizing that EF-corrected U-ExM measurements should be interpreted primarily as relative measurements and should not be treated as direct substitutes for measurements obtained from non-expanded samples without caution. We also note that manual measurement of BB/MC width and other protein signal dimensions was already described in the Methods section under "Protein signal length, width, and distance."

Line 505: Another technical consideration is that dimensional measurements obtained from U-ExM images depend on expansion factor correction. Therefore, EF-corrected values should be interpreted primarily as relative measurements among samples processed and imaged under standardized conditions, rather than as direct substitutes for measurements obtained from non-expanded specimens.

Detailed comment 15: Fig. 1C: Please remark on the CEP290 labeling in the "bulge region" of NHP rods. Is this high background or real labeling? Also, please remark on the distance of the DC from the MC throughout the data. There appears to be wide variability; the published centriol-centriole distance from mouse rod cryoET data in Gilliam et al. (2012) is ~500 +/- 100 nm. In some U-ExM examples throughout these figures the MC-DC distance noticeably exceeds that distance.

Response: Regarding the CEP290 signal in the bulge region of the NHP rod shown in the original Fig. 1C, we consider that the signal could be interpreted as nonspecific background rather than definitive CEP290 labeling in this region. To avoid confusion, we have replaced the NHP rod panels in Fig. 1C with images showing lower nonspecific background and clearer CEP290 labeling of the CC.

Regarding the distance between the BB (MC) and DC, we agree that some examples, particularly in NHP rods, show a greater apparent separation between the BB and DC than expected from the mouse rod cryo-ET measurements reported by Gilliam et al. (2012). We had noticed this feature during image analysis, but did not emphasize it in the original manuscript because it was most apparent in NHP rods and was not consistently observed in human rods. Thus, unlike the CC length, proximal OS axoneme/bulge morphology, or cone DC elongation, this feature did not show a consistent primate-associated pattern across NHP and human samples.

We also note that the functional significance of BB-DC spacing in mature PSC and primary cilia remains unclear, and this parameter was not the focus of the present study. Because we did not perform formal quantitative analysis of BB-DC distance, we have avoided drawing mechanistic conclusions from this observation. However, in response to the reviewer's comment, we have added a brief note to the Results section stating that some NHP rods showed an apparently larger BB-DC separation than other photoreceptor groups, while emphasizing that this observation was qualitative and not further interpreted.

© 2026. Published by The Company of Biologists under the terms of the Creative Commons Attribution License (https://creativecommons.org/licenses/by/4.0/).

Line 150: We also noted that NHP rods showed a larger apparent separation between the BB and DC than the other photoreceptor groups (**Fig. 1C, G**), although BB–DC distance was not formally quantified and was not further interpreted.

Detailed comment 16: Line 130, Fig. 1 F: Please confirm in the text or figure legend that CC axoneme width measurements were calculated from the tubulin immunolabeling (anti-acTub).

Response: We have revised the Fig. 1E, F legend to clarify that CC length was measured from CEP290 immunolabeling, whereas CC axoneme width was measured from AcTub immunolabeling.

Detailed comment 17: Line 142: A brief description of the RP1 and LCA5 proteins should be provided.

Response: We have revised the text to briefly describe RP1 as a photoreceptor axoneme-associated protein and LCA5/lebercilin as a photoreceptor cilium-associated protein before introducing them as markers of the distal axoneme and bulge region, respectively.

Line 154: To define the structural organization of the distal CC/proximal OS axoneme region, we next labeled retinitis pigmentosa 1 protein (RP1), a photoreceptor axoneme-associated protein, and Leber congenital amaurosis 5 protein (LCA5/lebercilin), a cilium-associated protein enriched in the bulge region (Takahashi et al., 2025). These proteins served as molecular markers of the distal axoneme and bulge region, respectively (**Fig. 2A, B**).

Detailed comment 18: Line 149: Terminology consistency is needed: the "distal PSC" likely means the OS or the OS axoneme.

Response: We replaced this wording with “the region distal to the CC end”.

Detailed comment 19: Figs. 2E-G have less value since they have no quantification associated with the images. Additionally, the NHS-ester stained OSs in Fig. 2F are distended and difficult to interpret. These images should be considered as supplemental views to the more informative longitudinal images already presented.

Response: We agree that, in the original manuscript, Fig. 2E–G was presented primarily as image-based morphological evidence and was not accompanied by quantification. We have therefore revised the text to qualify these observations as qualitative morphological observations and to avoid implying that these panels provide quantitative evidence for differences in OS axoneme/bulge length or width.

However, we respectfully disagree that the axial-view images have limited value or should be considered merely supplemental to the longitudinal images. The purpose of Fig. 2E,F is not simply to repeat the lateral-view observations, but to show the cross-sectional morphology of the proximal OS axoneme/bulge region at defined positions distal to the CC end. This information is important because the proximal OS axoneme/bulge region does not appear to be radially symmetric in all photoreceptor groups. In canine rods, the bulge region showed a relatively uniform morphology across lateral and axial views. By contrast, canine cones, NHP rods, and NHP cones showed more variable appearances in lateral-view images, as can be appreciated by comparing Fig. 1C,G and Fig. 2A,B. We interpret this variability as reflecting, at least in part, a more elliptical or anisotropic cross-sectional morphology of the proximal OS axoneme/bulge region. In lateral views, the apparent width of such a structure can depend on the orientation of the photoreceptor relative to the imaging plane and whether the long or short axis is visualized. Therefore, axial-view images provide complementary information that cannot be fully inferred from longitudinal views alone.

In response to the reviewer’s concern, we have revised the Results text to state more cautiously that the proximal OS axoneme/bulge morphology was more pronounced based on qualitative morphological observations, rather than presenting this as a quantified dimensional difference. We have also removed the original Fig. 2G, which we agree was less informative without accompanying quantification.

Regarding the NHS-ester-stained OS profiles in Fig. 2F, we understand the reviewer’s concern that

© 2026. Published by The Company of Biologists under the terms of the Creative Commons Attribution License (https://creativecommons.org/licenses/by/4.0/).

these structures may appear distended and difficult to interpret. However, we do not use Fig. 2F to draw conclusions about OS size or OS dimensions. In this panel, NHS-ester staining was used only as a pan-protein morphological reference to indicate the surrounding photoreceptor structure. Conclusions regarding IS/OS size are based on other data, including the non-expanded WGA/PNA images added in response to Reviewer #1 Comment 2, and not on the NHS-ester signal in Fig. 2F.

Thus, we have retained the axial-view images in the main figure because they provide important qualitative cross-sectional information, but we have revised the wording, removed the less informative panel, and clearly distinguished these qualitative morphological observations from quantitative measurements.

Line 160: In addition, qualitative morphological observations of RP1 and LCA5 distribution indicated that NHP photoreceptors possess a more pronounced bulge morphology than canine photoreceptors. To examine this region in more detail, we performed axial-view imaging of the region distal to the CC end **(Fig. 2E, F)**. In canine photoreceptors, the bulge morphology appeared more pronounced in cones than in rods, consistent with the quantitative findings in previous report (Takahashi et al., 2025). In NHP photoreceptors, both rods and cones displayed more pronounced bulge morphology than canine rods, with NHP cones showing the most pronounced morphology among all groups. In addition, the bulge region in canine cones and NHP photoreceptors appeared more elliptical than in canine rods **(Fig. 2F)**.

Detailed comment 20: Line 157: The authors must qualify that the larger OS axoneme bulge difference conclusion finding is qualitative or more concisely state that the OS axoneme bulge (widening) was more pronounced based strictly on morphological observations.

Response: We have revised the statement to clarify that the difference in the proximal OS axoneme/bulge region was based on qualitative morphological observations rather than quantitative width measurements. Specifically, we now describe this feature as “a more pronounced bulge morphology.”

Detailed comment 21: Line 169: Noticeable morphological variation is suggested in the human imaging data of Fig. 3A, but this is not apparent and should be discussed. Better yet, please provide lower magnification views for the assessment of the overall tissue quality.

Response: We agree with the reviewer that the inter-replicate variability was not sufficiently apparent from the representative images in Fig. 3A alone. Our intention was not to imply that Fig. 3A by itself fully demonstrated this variability, but rather to introduce the inter-replicate differences that were further supported by the quantitative analyses shown in Fig. 3B and Fig. 3D.

We have therefore revised the text to cite Fig. 3A, B and D at this point and to state more precisely that U-ExM analysis revealed inter-replicate variability in PSC morphology and quantitative measurements between the two human biological replicates.

Line 183: U-ExM analysis revealed inter-replicate variability in PSC morphology and quantitative measurements between the two human biological replicates (**Fig. 3A, B, D**).

Detailed comment 22: Line 181: The authors should reference the Moye et al. (2026) Am J Hum Genet paper (PMID: 41742423), which shows a particularly striking elongated daughter centriole in an expanded human cone (Fig. 3C). Many readers will be interested in comparing the data.

Response: We were aware of the expanded human cone image shown in Moye et al. (2026), as well as a related earlier report by the same first author (Moye et al., 2025, PMID: 40704549) in which an elongated daughter centriole is also visible in human cone photoreceptors. However, in these studies, the elongated daughter centriole morphology in human cones was not specifically described, analyzed, or discussed, presumably because this feature was not the main focus of those papers.

For this reason, in the original manuscript, we cited Mercey et al. (2024) as the primary reference, because that study explicitly commented on structural differences between human rod and cone PSC axoneme-associated structures. Nevertheless, we agree that the images in Moye et

© 2026. Published by The Company of Biologists under the terms of the Creative Commons Attribution License (https://creativecommons.org/licenses/by/4.0/).

al. (2025) and Moye et al. (2026) provide useful visual points of comparison for readers. We have therefore added these two papers to the Discussion as supporting references, while retaining Mercey et al. (2024) as the principal reference for the interpretation of elongated daughter centrioles in human cones.

Detailed comment 23: Line 192: Please qualify these "bulge" region observations as just that: morphological observations. Also, the "bulge" in the axoneme appears "wider" or more "distended." The term "extended" is not as clear, and the suggestion of "greater expansion" is potentially confusing for obvious reasons.

Response: We have revised this section to clarify that the bulge-region observations are qualitative morphological observations rather than quantitative measurements of axonemal width or length. We have also replaced the terms “extended” and “greater expansion,” which could be ambiguous in the context of expansion microscopy, with more specific morphological descriptions such as “more pronounced bulge morphology” and “wider axonemal bulge morphology.”

Line 207: Based on qualitative morphological observations, the proximal OS axoneme/bulge region appeared more pronounced in both human rods and cones than in canine photoreceptors, and this feature was particularly evident in cones, which showed a wider axonemal bulge morphology similar to that observed in NHP cones (**Fig. 3F**). Together, these findings indicate that the molecular architecture of the PSC axoneme differs between canine and primate photoreceptors, whereas human photoreceptors show several structural similarities to NHP photoreceptors. At the same time, the fundamental rod–cone differences in PSC architecture were maintained across all species, with cones consistently exhibiting shorter CCs, longer DCs, and more pronounced bulge morphology than rods.

Detailed comment 24: Fig. 3G should be removed due to the lack of quantification proving the OS axoneme is wider in cones. The morphological conclusion is apparent (and valid) based on the images themselves.

Response: We respectfully disagree that Fig. 3G should be removed. Fig. 3G is not presented as quantitative evidence for OS axoneme width. Rather, it is a schematic summary of the qualitative morphological features observed in the representative images shown in Fig. 2 and 3. Importantly, the reviewer also notes that the morphological conclusion is apparent and valid based on the images themselves. If the image-based morphological conclusion is valid, we believe that summarizing this conclusion schematically is appropriate, provided that the schematic is clearly described as a qualitative summary rather than as a quantitative analysis.

We also note that Fig. 3G serves the same purpose as the other summary schematics in the manuscript, including Fig. 4G and Fig. 5F. These panels synthesize qualitative morphological observations across multiple markers, species, and rod/cone-subtypes and are included to help readers follow the complex structural comparisons presented in the study. Removing Fig. 3G solely because it summarizes qualitative rather than quantitative observations would therefore be inconsistent with the role of these schematic summary panels throughout the manuscript. The schematic does not replace the primary image-based evidence, but provides an interpretive guide to the observations that are directly shown in the corresponding image panels. We have therefore retained Fig. 3G. In response to Reviewer#1 Comment 6, we moved original Fig. 3G to revised Fig. 2G.

Detailed comment 25: Line 205: Curiously, the region that is called the IS/OS boundary here is termed the IS/OS junction in the referenced Sahly et al. 2012 paper. Why the distinction?

Response: We used “boundary” as a descriptive anatomical term to indicate the interface between the inner segment and outer segment in fluorescence/U-ExM images. This terminology has also been used in previous photoreceptor studies, including studies of calyceal process organization **(PMID: 38477343)**. In addition, because “IS/OS junction” was historically used in the OCT literature to describe an outer retinal reflective band that is now commonly referred to as the ellipsoid zone, we initially used “boundary” to avoid potential ambiguity.

Nevertheless, we agree that maintaining consistency with Sahly et al. (2012) is preferable in this

© 2026. Published by The Company of Biologists under the terms of the Creative Commons Attribution License (https://creativecommons.org/licenses/by/4.0/).

context. We have therefore revised “IS/OS boundary” to “IS/OS junction” in the relevant text.

Detailed comment 26: Line 207: Please provide an appropriate reference for this statement about rod calyceal processes.

Response: We have added appropriate references supporting the statement that calyceal processes are absent or vestigial in rodent photoreceptors. In particular, Sahly et al. reported that typical calyceal processes were not detected in mouse photoreceptors. We also cite additional literature summarizing that calyceal processes are largely absent in nocturnal rodents, with only occasional vestigial or non- classical protrusions reported in mouse photoreceptors (Sharkova et al., 2023).

Detailed comment 27: Line 213: It seems like an overstatement that NHS-ester counterstaining provides "clear delineation of periciliary subcompartments." Perhaps it "helps" determine the subcompartment locations?

Response: We have rephrased this sentence as follows.

Line 226: To visualize representative components of USH1 and USH2 protein complexes, we performed immunolabeling for protocadherin-15 (PCDH15) and Whirlin, respectively, in combination with NHS-ester counterstaining, which provided a morphological reference for identifying the approximate locations of periciliary subcompartments.

Detailed comment 28: Line 226: What are "singlets"? Are they branches from the "pillars"? The conclusion of the line scans in Fig. 4D needs more explanation. Are these same patterns found in other rods?

Response: As described in our response to Reviewer #1 Comment 7 and Reviewer #2 Major Comment 5, we have revised these descriptions to avoid unsupported terminology such as “singlets” and to improve the explanation of Fig. 4D. We have also added serial z-section images in Supplementary Fig. S6 to support the revised interpretation of Whirlin-positive organization in additional rods.

Detailed comment 29: Line 236: Based on the examples in Fig. 4F, the actin staining seems comparable between canine and NHP rods. Therefore, please reconsider terms like "prominent" and "robust." The morphological observation of the alignment of the actin streaks with the NHP PCDH15 is accurate.

Response: We have removed stronger descriptive terms such as “prominent” and “robust” when referring to actin staining intensity.

Detailed comment 30: Line 242: "Weakly developed" is a vague term. What is the meaning? Do canine rods have calyceal processes? Perhaps they are smaller and more difficult to capture with immunofluorescence?

Response: Our intention was not to definitively distinguish between complete absence of calyceal processes and the presence of very limited or poorly developed calyceal process-like structures in canine rods. Because calyceal processes are ultrastructural features, this distinction cannot be resolved solely by PCDH15 and actin immunolabeling.

We have therefore revised the text to state more precisely that canine rods did not show clear PCDH15/actin-associated molecular features comparable to the well-defined calyceal processes observed in NHP rods. We also avoid making a definitive conclusion as to whether canine rods completely lack calyceal processes or possess only very limited calyceal process-like structures.

Line 256: Furthermore, canine rods did not show clear PCDH15/actin-associated molecular features comparable to the well-defined calyceal processes observed in NHP rods. These findings suggest that calyceal processes are absent or, if present, are very limited in canine rods. By contrast, NHP rods displayed prominent PCDH15-positive, actin-associated profiles extending along the basal OS, consistent with well-defined calyceal processes (**Fig. 4G**).

© 2026. Published by The Company of Biologists under the terms of the Creative Commons Attribution License (https://creativecommons.org/licenses/by/4.0/).

Line 459: suggesting either absence or very limited development of calyceal process-like structures in this photoreceptor population.

Detailed comment 31: Line 255: Here, the "pillar-like" elements are called "paired doublet-like" elements. Much more consistency and better clarity about these novel structures are needed.

Response: As described in our response to Reviewer #2 Major Comment 5, we have revised the terminology used for these Whirlin-positive structures to improve consistency and clarity.

Detailed comment 32: Line 277: Clarification needed: Only 1 rod in Fig. 6A shows calyceal-like process PCDH15 labeling.

Response: We understand the reviewer's concern that axially oriented, calyceal process-like PCDH15 labeling appears limited in the single optical section images of human replicate 1 rods compared with human replicate 2 rods.

However, the PCDH15-positive calyceal process-like signal in human replicate 1 was not limited to a single rod profile. As shown in the 3D-rendered panels in Fig. 6A and Fig. 6B, weak but discernible axially oriented PCDH15-positive structures were detected in human replicate 1 rods. Because the PCDH15 signal was weaker in human replicate 1 than in human replicate 2, this pattern is difficult to appreciate in some single optical sections. In Fig. 6A, the single optical sections were selected primarily to show the bilateral Whirlin-positive structures, and therefore do not fully capture the three- dimensional distribution of the PCDH15-positive signal.

To avoid ambiguity, we have added one sentence in Results section to clarify that the PCDH15-positive calyceal process-like structures in human replicate 1 were weak and were more clearly appreciated in the 3D-rendered images. This clarification does not change our interpretation that human rods show PCDH15-positive periciliary/calyceal process-like organization, although the signal was weaker in human replicate 1 than in human replicate 2.

Line 293: This axially oriented PCDH15 signal was weaker in human replicate 1 than in human replicate 2 and was more clearly appreciated in 3D-rendered images than in individual optical sections.

Detailed comment 33: Line 293: "Highly recent" is a vague term. What is the meaning?

Response: We have rephrased this sentence as follows.

Line 310: Because this finding was first reported in a recent study

Detailed comment 34: Line 301: Please clarify: Do all the rods in the human donor replicate 1 retina show aIS-like structures?

Response: We assume that the reviewer is referring to the human donor 2 retina, in which aIS-like structures were observed. In the human donor 2 retina, aIS-like structures were not observed in all rod photoreceptors. Even among rods with aIS-like extensions, their morphology was variable, ranging from relatively long OS-apposed extensions to much more limited profiles, as represented in the upper and lower donor 2 panels in Fig. 7A.

We attempted to estimate the proportion of rods with aIS-like structures in donor 2. However, this donor retina had a substantially longer interval between death and fixation and showed poorer overall tissue preservation than donor 1. For this reason, we judged that broad sampling across the donor 2 retina would not provide a reliable quantitative estimate of aIS prevalence. Instead, imaging was restricted to relatively well-preserved retinal regions suitable for structural interpretation.

We also note that the biological interpretation of such a prevalence estimate would remain limited, because Lewis et al. (2025) did not report whether all rods uniformly possess aIS structures, the prevalence of aIS-positive rods, or the degree of morphological variability among

© 2026. Published by The Company of Biologists under the terms of the Creative Commons Attribution License (https://creativecommons.org/licenses/by/4.0/).

individual rod aIS structures. Therefore, even if we obtained a preliminary estimate of aIS prevalence in our donor 2 sample, we could not directly compare this value with a reliable published reference dataset. We have revised the Results and Discussion to clarify that the aIS-like structures observed in donor 2 were variable and were not present in all rod profiles.

Line 322: The extent of this aIS-like structure varied markedly among rods, and clear aIS-like extensions were not observed in all rod profiles in human replicate 2.

Line 477: In donor 2, these aIS-like extensions were not observed in all rod profiles, and their morphology varied from relatively long OS-apposed extensions to more limited profiles. Because the prevalence of aIS-positive rods and the extent of cell-to-cell morphological variability were not defined in the previous study (Lewis et al., 2025), further analysis will be required to determine how consistently this architecture is present in human rods.

Detailed comment 35: Line 307: No tubulin scaffold is reported in the aIS-like structure; however, there appears to be beta-tubulin staining in human replicate 2 in Fig. 7A (actually right where the arrowhead is pointing). Please show the beta-tubulin channel only. What does this staining represent?

Response: Please refer also to our response to Reviewer #1 Comment 11. We agree that β-tubulin staining is present within the aIS-like structure in human replicate 2. We have therefore revised our interpretation and removed the statement implying that tubulin signal was absent from the aIS-like structure. We also added **Supplementary Fig. S8**, which includes single-channel β-tubulin and α- tubulin images. These data show that the aIS-like structures contain tubulin-positive signals, but these signals appear variable and uneven, resembling the broader IS microtubule network rather than forming a scaffold unique to the aIS-like region.

Detailed comment 36: Line 313: The term "abnormality" should be reconsidered.

Response: We have revised the relevant paragraphs as follows.

Line 331: replicate 1 again lacked any prominent morphological variation

Detailed comment 37: Line 330: Considering that the rootlet is "tethered" to the BB (see Wensel et al. 2021 and Sánchez-Bellver et al. 2021), it more logically extends down from the BB into the IS cytoplasm.

Response: We have revised the relevant paragraphs as follows.

Line 351: from the BB toward the basal IS

Detailed comment 38: Line 335: Without rootlet measurements, please qualify the conclusions as "apparent," "appeared," or based on "morphological observation."

Response: We have revised the relevant paragraphs as follows.

Line 355: By contrast, based on qualitative morphological observations of rootletin-labeled structures, ciliary rootlets in NHP and human retinas appeared to exhibit structural features distinct from those in canine retina. In rods, both NHP and human photoreceptors retained a generally straight rootlet architecture resembling that of canine rods, but these structures appeared thicker than canine rod rootlets and appeared to consist of multiple filamentous elements bundled together into a single rootlet structure (**Fig. 8A**). Cone photoreceptors showed an even more apparent species-dependent difference. Unlike canine cones, in which the rootlet terminal portions remained connected in a continuous arc-like configuration, NHP and human cones appeared to show separated rootlet termini extending as two or three thin fibers. These fibers did not appear to form a continuous arc-like terminal structure and instead appeared to extend into the intermediate region of the IS (**Fig. 8B; Supplementary Fig. S9**). Thus, although the overall rod- and cone-specific configuration appeared to be preserved across species, the detailed morphology of the rootlet appeared to differ between canine and primate photoreceptors.

© 2026. Published by The Company of Biologists under the terms of the Creative Commons Attribution License (https://creativecommons.org/licenses/by/4.0/).

In contrast to the PSC axoneme and the aIS-like structure, no obvious difference in ciliary rootlet morphology was observed between the two human donor samples by qualitative morphological observation (**Fig. 8A, B**). Thus, the apparent species-specific features of human rootlets were relatively consistent across the human specimens examined. Together, these findings suggest that ciliary rootlet architecture shows clear rod–cone differences in all three species, while the specific morphological features of rod and cone rootlets appear to differ between canine and primate photoreceptors.

Detailed comment 39: Line 361: This sentence is redundant and difficult to read. Consider removing it to get to the next point.

Response: We have removed this sentence.

Detailed comment 40: Line 372: "Comprehensive inclusion" reads like flowery jargon that should be reconsidered.

Response: We have rephrased this sentence as follows.

Line 396: Moreover, through systematic subcompartment-level analysis of the PSC in rods and cones, we demonstrate that differences in PSC organization among species and between rods and cones are more extensive and nuanced than previously appreciated.

Detailed comment 41: Line 405: Is an EM paper being referenced here, or is this referencing unpublished observations?

Response: This statement was not intended to refer to unpublished observations. Rather, we intended to indicate that, to our knowledge, calyceal processes in mature canine cone photoreceptors have not been definitively documented by prior electron microscopy studies.

Because this statement refers to the absence of a definitive published EM demonstration, rather than to a specific EM study, we did not cite an EM paper at this point. To avoid ambiguity, we have revised the sentence to make clear that this is based on our interpretation of the published literature and not on unpublished data.

Line 462: However, canine cones displayed a clear calyceal process-like molecular organization, although, to our knowledge, calyceal processes in canine cones have not been definitively documented by prior electron microscopy studies.

Detailed comment 42: As detailed above, the functional implications of the major findings in this paper should be included in the manuscript.

Response: Please refer to our response to Reviewer #2 Major Comment 1.

Detailed comment 43: Line 496: For the sake of readers unfamiliar with the ExM technique, please provide more details for "post-expansion immunohistochemistry, counterstaining, and second-round expansion." Also, how were gels mounted for imaging? How long were the imaging periods? Expansion potentially continues in a water-based mountant during long imaging periods. Could this be an important technical consideration with this method?

Response: We have expanded the Methods section to describe the post-expansion immunohistochemistry, counterstaining, second-round expansion, gel mounting, and imaging conditions in greater detail.

Line 571: After first-round expansion, gels were equilibrated in 1× PBS and then incubated in blocking buffer for 30 minutes. The gels were incubated with primary antibodies diluted in blocking buffer overnight, followed by incubation with fluorophore-conjugated secondary antibodies diluted in blocking buffer for 2 hours at room temperature. When indicated, NHS-ester staining was performed after immunolabeling as a pan-protein counterstain. After the labeling procedure, gels were re- expanded in excess distilled water for the second-round expansion and kept at 4°C until imaging.

© 2026. Published by The Company of Biologists under the terms of the Creative Commons Attribution License (https://creativecommons.org/licenses/by/4.0/).

Line 581: U-ExM samples were imaged using the 63× water-immersion objective (NA 1.20). For imaging, expanded gels were transferred to poly-D-lysine-coated glass-bottom dishes. The dishes were kept humidified during image acquisition to prevent gel drying and shrinkage, and imaging of each gel was completed within 30 minutes.

Detailed comment 44: Line 639, Figure 1 Legend (and throughout): Please confirm that all imaging data are U-ExM.

Response: In the original manuscript, all imaging data were obtained from U-ExM samples. In the revised manuscript, the only exception is Supplementary Fig. S3, which shows non-expanded retinal sections stained with WGA and PNA. To avoid ambiguity, we have added the statement "All microscopy images in this figure are U-ExM images." to the relevant figure legends. We have also explicitly stated in the Supplementary Fig. S3 legend that these images were obtained from non-expanded retinal sections.

Detailed comment 45: Line 644, MIP as an abbreviation for maximum intensity projection, is unnecessary and could be confused with its use as an abbreviation for microtubule inner protein by cilia/flagella experts.

Response: We have removed the abbreviation "MIP" and now spell out "maximum-intensity projection" throughout the manuscript and figure legends.

Detailed comment 46: Line 679: Describing NHS-ester staining of the "photorecepotor soma" is unusual. The staining is either OS membranes or the apical IS.

Response: We agree that describing the NHS-ester signal as labeling the "photoreceptor soma" was imprecise in this context, and we have revised this wording. However, NHS-ester is not intended to label only OS membranes or the apical IS. Rather, NHS-ester broadly reacts with primary amine- containing proteins and was used here as a pan-protein counterstain to provide morphological context for surrounding photoreceptor structures. We have therefore revised the figure legends to describe NHS-ester staining as a pan-protein morphological reference rather than as a marker of the photoreceptor soma.

<u>Second decision letter</u>

MS ID#: jcs.265080R1

MS TITLE: Cross-species molecular mapping of the photoreceptor sensory cilium and periciliary structures identifies conserved and species-specific architectural features

AUTHORS: Kei Takahashi; Mayuna Obayashi; Raghavi Sudharsan; William A. Beltran

ARTICLE TYPE: Research Article

Dear Dr Beltran,

We have now reached a decision on the above manuscript.

As you will see, the reviewers gave favourable reports but raised some critical points that will require amendments to your manuscript. I hope that you will be able to carry these out because I would like to be able to accept your paper, depending on further comments from reviewers.

<u>Reviewer 1</u>

The authors successfully addressed critical points raised in the previous cycle of review. These data are of interest to specialists studying photoreceptor anatomy and function.

© 2026. Published by The Company of Biologists under the terms of the Creative Commons Attribution License (https://creativecommons.org/licenses/by/4.0/).

Reviewer 2

All major critiques were addressed in the revised manuscript and response to reviewers. The revised manuscript is now more comprehensive and is impactful work. I commend the authors for their commitment to such an intensive imaging study.

Comment on revised manuscript:

Fig. 1C is not described in the Results section, nor are POC5 nor the POC5 immunolabeling images in Fig. 1C.

Second revision

Author response to reviewers' comments

Reviewer #1

The authors successfully addressed critical points raised in the previous cycle of review. These data are of interest to specialists studying photoreceptor anatomy and function.

Response: We greatly appreciate the reviewer's positive assessment of the revised manuscript and the constructive feedback provided throughout the review process.

Reviewer #2

All major critiques were addressed in the revised manuscript and response to reviewers. The revised manuscript is now more comprehensive and is impactful work. I commend the authors for their commitment to such an intensive imaging study.

Response: We sincerely appreciate the reviewer's encouraging assessment of the revised manuscript and valuable suggestions, which have helped us further improve the clarity and completeness of the work.

Comment on revised manuscript: Fig. 1C is not described in the Results section, nor are POC5 nor the POC5 immunolabeling images in Fig. 1C.

Response: We thank the reviewer for pointing out this omission. We have now included POC5 among the additional CC-associated markers described in the Results section and added the corresponding citation to Fig. 1C.

Line 137: These rod-cone and species-dependent differences in CC length were further supported by labeling with protein of centriole 5 (POC5), a component of the CC inner scaffold (**Fig. 1C**). Additional support was provided by Y-link-associated labeling with spermatogenesis associated 7 (SPATA7) and glutamylation signals located outside the ciliary axoneme (**Fig. S4**).

Third decision letter

MS ID#: jcs.265080R2

MS Title: Cross-species molecular mapping of the photoreceptor sensory cilium and periciliary structures identifies conserved and species-specific architectural features

Authors: Kei Takahashi; Mayuna Obayashi; Raghavi Sudharsan; William A. Beltran

Article Type: Research Article

Dear Dr Beltran,

I am happy to tell you that your manuscript has been accepted for publication in Journal of Cell Science, pending standard publication integrity checks.

© 2026. Published by The Company of Biologists under the terms of the Creative Commons Attribution License (https://creativecommons.org/licenses/by/4.0/).
